# Evolution of insect proteomes: insights into synapse organization and synaptic vesicle life cycle

**DOI:** 10.1186/gb-2008-9-2-r27

**Published:** 2008-02-07

**Authors:** Chava Yanay, Noa Morpurgo, Michal Linial

**Affiliations:** 1 Department of Biological Chemistry, Institute of Life Sciences, Givat Ram Campus, Hebrew University of Jerusalem, Jerusalem 91904, Israel

## Abstract

**Background:**

The molecular components in synapses that are essential to the life cycle of synaptic vesicles are well characterized. Nonetheless, many aspects of synaptic processes, in particular how they relate to complex behaviour, remain elusive. The genomes of flies, mosquitoes, the honeybee and the beetle are now fully sequenced and span an evolutionary breadth of about 350 million years; this provides a unique opportunity to conduct a comparative genomics study of the synapse.

**Results:**

We compiled a list of 120 gene prototypes that comprise the core of presynaptic structures in insects. Insects lack several scaffolding proteins in the active zone, such as bassoon and piccollo, and the most abundant protein in the mammalian synaptic vesicle, namely synaptophysin. The pattern of evolution of synaptic protein complexes is analyzed. According to this analysis, the components of presynaptic complexes as well as proteins that take part in organelle biogenesis are tightly coordinated. Most synaptic proteins are involved in rich protein interaction networks. Overall, the number of interacting proteins and the degrees of sequence conservation between human and insects are closely correlated. Such a correlation holds for exocytotic but not for endocytotic proteins.

**Conclusion:**

This comparative study of human with insects sheds light on the composition and assembly of protein complexes in the synapse. Specifically, the nature of the protein interaction graphs differentiate exocytotic from endocytotic proteins and suggest unique evolutionary constraints for each set. General principles in the design of proteins of the presynaptic site can be inferred from a comparative study of human and insect genomes.

## Abstract 2

A comparative study of human versus insects sheds light on the composition and assembly of protein complexes in the insect synapse.

## Background

The completion of the *Drosophila malengaster *genome in the year 2000 provided the first glimpse at the make-up of animals with a complex nervous system [1,2]. The availability of several genomes from insects, representing an evolutionary distance of 250 to 300 million years, provided a unique opportunity to evaluate the foundation of a functional synapse [3]. With many additional animal genomes now available, including those of primates, marsupials, fish and birds, a molecular correlation between genes and brain complexity is being actively sought [4,5].

*Drosophila *has been used for decades as a model in which to study synapse formation, embryogenesis, development, and neurogenesis [6]. A combination of biochemical, cell biologic, genetic, morphologic, and electrophysiologic studies have unravelled the molecular mechanisms of synaptic vesicle exocytosis and endocytosis in the fly [7,8] and compared these with the corresponding mechanisms in vertebrates [9]. In all neurons, communication across the synapse is mediated by neurotransmitter release from synaptic vesicles. Because the entire process may take only a fraction of a millisecond (in fast releasing synapses), additional processes ensure the priming, targeting, and docking of synaptic vesicles at the active zone [10].

Only the basic mechanism of vesicle fusion is shared between yeast and human [11]. Specifically, the minimal set of SNARE (Soluble NSF Attachment protein [SNAP] REceptor) functions is a unified mode of vesicle trafficking. The proper targeting and docking of synaptic vesicles is mediated by a cognate interaction between vesicular SNAREs (v-SNAREs) and target membrane SNAREs (t-SNAREs). The genuine synaptic vesicle protein associated membrane protein (VAMP; also called synaptobrevin) acts as v-SNARE, whereas the presynaptic membrane proteins syntaxin and SNAP-25 (SNAP of 25 kDa) are t-SNAREs. The multimeric ATPase NSF (*N*-ethylmaleimide sensitive fusion ATPase) is later recruited to the SNARE complex by SNAPs [12] and acts to break the extremely stable SNARE complex, thus reactivating the individual SNAREs for future fusion events. Unlike yeast secretion and vesicle trafficking, synaptic vesicle fusion in the presynaptic structure requires a large body of regulators to ensure the spatial and temporal resolution of neurotransmitter release [13].

Regulators of the SNAREs are numerous, and many of them are conserved throughout evolution. Examples are the Rabs and their direct regulators [14]. Specifically, Rab3, Rab5, Rab27, and Rab11 regulate vesicle transport, docking, and exocytosis of synaptic vesicles [15]. Many of the other Rabs function in membrane trafficking in general and are strongly conserved [16,17].

Recently, the composition and the stoichiometry of proteins and lipids of synaptic and transport vesicles from rat brain were presented [18]. Based on Mass spectrometry (MS) proteomics technology, about 80 proteins were identified. The synaptic role of many of these proteins was already established, mainly based on the genetics of model organisms such as *Drosophila melanogaster *and *Caenorhabtidis elegans *[2]. Schematically, the proteins of the synaptic vesicles are associated with the following functional groups: organizers and cytoskeletal scaffold proteins; transporters and channels; sensors and signal transduction proteins; priming, docking, and fusion apparatus [19,20]; endocytotic and recycling machinery [7,21-23]; and linkers between the presynaptic and postsynaptic membranes [2].

In addition, scaffolding proteins are critically important during the development and shaping of new synapses [24]. These proteins are a combination of adhesion, cytoskeleton, and signaling proteins. The specificity of neurons in the central nervous system (CNS) is primarily defined by the composition of receptors, transporters, and ion channels in the presynaptic and postsynaptic density (PSD) structures [25]. In addition to their role in neuronal transmission through ion channels, PSD proteins are essential in establishing a protein network that bridges the cytoskeleton to the extracellular matrix [2].

Herein, we focus on the basic function of the synapse, and specifically the trafficking, exocytosis, and endocytosis of synaptic vesicles, and analyze it in molecular terms. We compiled a list of 120 gene prototypes, called 'PS120', which comprises the core set of proteins associated with synaptic vesicles and presynaptic structures. This list includes components of the SNARE complex and their regulators, as well as components of the trafficking and organization apparatus of the active zone. In comparison with humans, there are many fewer paralogous genes in the four insects whose genome sequence has been completed (namely fly, mosquito, honeybee, and beetle). This comparative view is instrumental for *in silico *genome annotations but it also exposes instances in which a specific gene or a regulation network is lost. We show that the number of protein-protein interactions in which a protein participates and the degree of sequence conservation from insects to human are positively correlated. The architectures of proteins responsible for processes in the synapse such as exocytosis and endocytosis differ markedly. We show that a systematic comparative genomics view of the fly, honeybee, mosquito, and beetle proteomes reveals general principles in the design of presynaptic structures.

## Results

### Evolutionary relationships among insects

Insects are an ancient group of animals, the first of which probably appeared 360 to 400 million years ago. Analyses of insect genomes and proteomes provide a unique opportunity to compare evolution between the model organism *D. melanogaster *and numerous additional insect genomes. The insects whose genomes were sequenced ensure coverage of a valuable phylogenetic breadth, spanning the fruit fly (*D. melanogaster*(, the honey bee (*Apis mellifera*), the red flour beetle (*Tribolium castaneum*), the mosquitoes (*Anopheles gambiae *and *Aedes aegypti*), the silk worm (*Bombyx mori*) and the wasp (*Nasonia vitripennis*). All together, about 330,000 protein sequences from insects are currently available in public protein databases, which already include 12 additional *Drosophila *genomes. A current list of insect genome projects is accessible in Additional data file 1. In the present study we refer only to representative genomes that are substantially divergent and include the beetle, honeybee, mosquito, and fly (with *D. melanogaster *being the reference). We focus on establishing a functional synapse whose molecular assembly governs learning and memory as well as the complex behavior of the organism.

### A catalog of presynaptic gene representatives from human and insects

We compiled an extended catalog of mammalian presynaptic proteins based on the detailed anatomy of the synaptic vesicle [18], data from functional annotations by Gene Ontology (GO) [26], and a manual collection of genes of presynaptic function [27]. This collection is compared with insect proteomes. A summary of the sequence conservation of each gene (a total of 120 representative genes) with the insect proteome is shown in Table 1. Analyzing this catalog (PS120 - presynaptic 120 genes) revealed that 50% are well conserved and have a sequence similarity in excess of 65% for most of the sequence. Among them, 60% are at a similarity level in excess of 75% for most of the sequence. Thus, the majority of proteins that participate in human presynaptic structures are extremely well conserved.

**Table 1:** Presynaptic protein prototypes

Number	Gene	Name	S	M
1	ADD2	β-Adducin	D	
2	AMPH	Amphiphysin 1	D	
3	AP2A1	AP-2 α-adaptin	A	
4	AP3D1	AP-3 δ-adaptor	A	
5	APBA1	Mint1	C	
6	APBA2	Adapter protein X11β	B	*
7	ARF1	ARF 1	A	
8	ARF6	ARF 6	A	
9	ARFGEF2	ARF-GEF 2	B	*
10	ARFIP2	Arfaptin	B	
11	ATP6V0C	ATPase 16 kDa	A	
12	BAIAP3	Bai1-associated 3	D	*
13	BET1	Bet 1 homolog	B	
14	BIN1	Bridging integrator 1	D	
15	BLOC1S1	Lysosome BLOC1	B	*
16	BSN	Bassoon	E	*
17	CACNA1A	CaV2.1	B	
18	CADPS	Caps	C	*
19	CALM2	Calmodulin	A	
20	CASK	Lin-2 homolog	B	
21	CLTC	Clathrin heavy chain	A	
22	CNO	Cappuccino	D	*
23	CNTNAP1	Neurexin 4	D	
24	CPLX2	Complexin 2	C	
25	DLG1	SAP 97	B	
26	DNAJC5	HSP40 homologue	B	
27	DNM1	Dynamin 1	A	
28	DOC2B	Double C2	C	
29	EHD1	Testilin	A	
30	EPN1	Epsin-1	C	
31	EPS15	EGF substrate 15	D	
32	ERC1	Rab6 interact CAST	D	
33	EXOC6	Exocyst 6	C	
34	EXPH5	Slp homolog	E	*
35	FLJ20366	Syntabulin	E	*
36	SNAP29	SNAP 29	D	
37	GAP43	GAP 43	E	*
38	GDI2	Rab GDI 2	B	
39	GMRP	P-selectin	D	
40	GOPC	CFTR-associated ligand	C	*
41	GOSR2	Membrin	C	
42	HGS	Hepatocyte TK subs	C	
43	ITSN2	Intersectin	D	
44	KIF1A	Kinesin family 1	B	
45	LAMP1	Lysosomal 1	D	
46	LIN7A	Mals-1	A	
47	LPHN1	α-Latrotoxin receptor	D	*
48	MSS4	Rabif	C	*
49	MUTED	Muted	D	*
50	MYRIP	Rab-Myosin 7A	E	*
51	NET2	Tetraspanin-12	C	
52	NLGN2	Neuroligin-2	D	
53	NRXN1	Neurexin 1	D	
54	NSF	NEM-sensitive fusion	B	
55	PACSIN1	PKC and CK substrate	C	*
56	PCLO	Piccolo	D	*
57	PICALM	PI-binding clathrin	C	
58	PIK4CA	P I4-kinase α	C	
59	PIP5K1C	PI-4P 5-kinase 1γ	B	
60	PLDN	Pallidin	D	*
61	PPFIA3	Liprin α 3	B	
62	PSCD1	Cytohesin-1	A	
63	PSCD2	Arno 2	B	
64	RAB27A	Rab27A	B	
65	RAB3A	Rab3A	A	
66	RAB3GAP	Rab3 GTPase	D	*
67	RAB3IL1	Rabin 3	C	*
68	RAB6IP1	Rab6 interacting 1	C	
69	RABAC1	YIP3 homolog	C	
70	RABGAP1	Rab GTPase	C	*
71	RALA	Ral	A	
72	RAPGEF4	Rap GEF 4	C	
73	SEC22B	Sec22-like	B	
74	RILP	Rab-interact	E	*
75	RIMBP2	RimS binding	D	
76	RIMS1	Rims	D	
77	RPH3A	Rabphilin 3A	C	
78	SALF	Stoned B	D	
79	SCAMP1	SCAMP37	C	
80	SCIN	Scinderin	C	*
81	SEPT5	Septin 5	B	
82	SH3GL1	Endophilin	C	
83	SIPA1L1	Signal-proliferation 1	D	
84	SLC17A7	VgluT1	C	
85	SNAP25	SNAP-25	B	
86	SNAP91	AP180	D	
87	SNAPA	SNAP	B	
88	SNAPAP	Snapin	C	*
89	SNIP	Snip	D	*
90	SNPH	Syntaphilin	E	*
91	SNX9	Sorting nexin	D	*
92	STX1A	Syntaxin	A	
93	STXBP1	n-Sec	B	
94	STXBP5	Tomosyn	C	
95	STXBP6	Amisyn	E	*
96	SV2A	SV glycoprotein 1	D	
97	SYBL1	Synaptobrevin-like	B	
98	SYN	Synapsin	C	
99	SYNGR1	Synaptogyrin	C	*
100	SYNJ1	Synaptojanin	C	
101	SYNPR	Synaptoporin	E	*
102	SYP	Synaptophysin	E	*
103	SYT1	Synaptotagmin	B	
104	SYT5	Synaptotagmin	B	
105	SYT9	Synaptotagmin	C	*
106	SYTL4	Granulophilin	C	*
107	SYTL5	Synaptotagmin-like 5	D	*
108	TMEM163	synaptic vesicle31	E	
109	TRAPPC1	Bet5 homolog	C	
110	TRAPPC4	Sybindin	B	
111	TXLNA	α-Taxilin	C	*
112	UNC13B	Munc-13	B	*
113	UNC13D	Unc-13 homolog	D	
114	VAMP2	VAMP	A	
115	VAPA	VAP33	C	
116	VAT1	VAT-1	C	*
117	VPS18	Vacuolar sorting 18	D	
118	VPS33B	Vps-33B	D	
119	VTI1B	Vti1	D	
120	YWHAQ	14-3-3 protein	A	

The 120 presynaptic representatives from human (PS120) are indicated by their official gene names. Sequence conservation between human and insect proteomes is indicated by A to E. Sequence similarity index (S) is divided into five levels marked: A = >75%, B = >65%, C = >50%, D = >35%, and E = <34%. In the 'M' columns, an asterisk indicates that the gene is absent from the public protein databases. Detailed information on PS120 is provided in Additional data files 2a,2b.

Most of the PS120 proteins belong to gene families, with some of the families being very large. For example, synaptotagmins and Rabs have numerous alternative spliced variants in addition to their large number of genes (17 and 60, respectively). For most instances, the size of the gene family in insects is smaller and on average is only 40% when compared with human. To exemplify this observation, we investigate the syntaxin family. There are 12 genes in human (and additional variants) that can be divided into subfamilies. The human subfamily of syntaxin 1, which functions as the t-SNARE in synaptic vesicle fusion (including Stx1, Stx2, Stx3, Stx4, and Stx11), is represented by only two genes in the fly (namely dStx1 and dStx4) [1] and in the other insects. However, in general, there are more gene variants that result from alternatively splicing events in the fly genome relative to the other insects.

A search of insect homologs for the PS120 clearly shows that even within the most conserved set between human and insects (60 genes), there are 12 genes for which there is no clear homolog in the current protein databases in at least one of the insect representatives (honeybee, beetle, mosquito, and fly). The same applies to about 30 additional proteins from the remainder of the PS120 gene list. Additional information on protein partners and protein length, and detailed information on the levels of sequence conservation is provided in Additional data files 2.

### Recovering missed annotation genes by comparative genomics

The completion of genomes for at least four insect representatives and the additional information from partially assembled genomes (Additional data file 1) makes it possible to revisit some of the apparently missed genes (Table 1 and Additional data file 2). Evidently, comparing related genomes enhances the quality of *in silico *genome annotations [28]. A search in the public non-redundant database revealed that about one-third of the PS120 homologous sequences were missing in at least one of the insect representatives (Table 1). Moreover, for a small number of genes, no homologs were detected in any of the insects. In cases in which significant sequence similarity in all four insect representatives is absent, we strongly argue that these genes are genuinely absent in insects. This is supported by a lack of significant similarity in additional fly genomes, and in the silkworm and the wasp genomes (Additional data file 3).

Additional data file 3 provides information on apparently missing genes that are not apparent from protein databases (see Materials and methods, below). For 70% significant similarity in the genome-assembled sequences was identified. This high similarity is often supported by the existence of an expressed mRNA. For a few genes, only limited evidence on transcription levels exists. More importantly, for 11 genes no homologs were detected in insects by searching protein data against translated insect genomes. Among these genes are growth-associated protein (GAP)-43, which is implicated in cytoskeleton and protein kinase C signaling during synapse establishment [29], and two large proteins that shape the cytoskeletal mesh at the active zone: bassoon (about 3,900 amino acids) and piccolo (about 5,100 amino acids) [30]. In addition, the SNARE regulator complexin 4, the syntaxin-tubulin binding protein syntabulin (FLJ20366), and SNAP-25-interacting protein are not detected in insects. Although most proteins of the synaptic vesicle membranes are strongly conserved, we were unable to detect SV31 (also called TMEM163; a genuine protein of the synaptic vesicle (SV) membranes) [31] or synaptophysin (one of the most abundant proteins in mammalian synaptic vesicles). Furthermore, no sequence similarity was noted for the syntaxin regulators amisyn (STXBP6) [32] and syntaphilin [33]. Syntaphilin, which has been implicated in regulation of exocytosis and endocytosis [34], is conserved from human to pufferfish and zebrafish but was lost in the branch of the frogs and insects. A borderline similarity to dynactin and α-liprin suggests that the function in cytoskeletal remodeling and in cell-matrix interactions may be taken over by other proteins. Interestingly, many of the genes that are not conserved from human to insects are functionally related to active zone architecture and specifically to the underlying cytoskeleton mesh of the synapse.

### Insights into the most conserved proteins of the exocytosis core complex

In the PS120 gene list, rather close conservation is evident between insect and human genes (measured by a similarity >75% throughout the sequence) for 16 genes. This small set includes the v-SNARE VAMP2, the t-SNARE syntaxin 1A, and a few small GTP proteins (Ral, Rab3A, ARF1, and ARF6). In addition, this set includes essential components of the endocytic machinery (dynamin 1, AP2, AP3, EHD1, and clathrin) and proteins that activate transduction pathways (calmodulin and 14-3-3). That the function of these gene products is indispensable was expected, but proteins that coordinate synaptic vesicles with the active zone are also included in this selected list, namely cytohesin-1 [35] and Mals-1 [36]. Both of these proteins share a function in determining the size of the readily releasable pool of synaptic vesicles and are critical for replenishing this pool.

**Figure 1:**
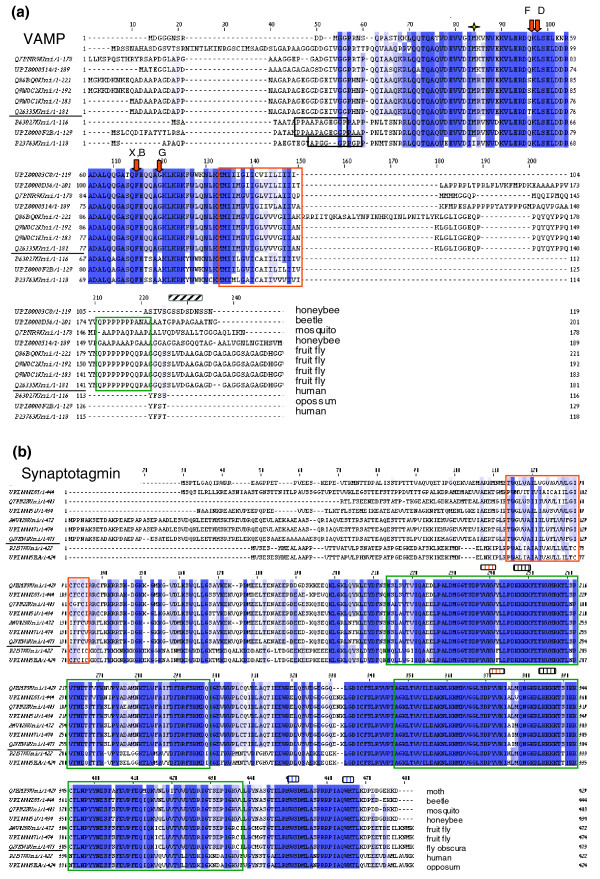
Multiple sequence alignments using for VAMP and synaptotagmin. The multiple alignment sequence (MSA) is performed using ClustalW. A graded blue color indicates the level of conservation among the representative sequences. Horizontal line in the protein accessions separates insect (top) and vertebrate (bottom) sequences. **(a) **Vehicle-associated membrane protein (VAMP; 11 sequences). The transmembrane domain is marked by a red frame. Proline rich domain in the amino-terminal of mammalian VAMP-2 is framed in gray and was implicated in synaptophysin regulation. Red arrows denote the identified tetanus toxin (X) and botulinum toxin (B, D, F, G) cleavage sites. The star indicates an essential biogenesis targeting signal. Stripped box indicates the calcium-calmodulin binding domain in mammalian VAMPs. A conserved low complexity region that is shared among all insects is enriched with stretches of Ala, Gly and Pro, and is marked by a green frame. Proteins (top to bottom): similar to CG17248 (iso A), honeybee; CG17248 (iso A), beetle; similar to VAMP, mosquito, CG17248 (iso A), honeybee; CG17248 (iso D), fruit fly; CG17248 (iso B), fruit fly; CG17248 (iso A), fruit fly; N-Syb, fruit fly, VAMP-2, human; VAMP-2, opossum; VAMP-1, human. **(b) **Synaptotagmin (nine sequences). Calcium sensor for neurotransmitter release that is characterized by two C2 domains (marked in green frames) and an amino-terminal transmembrane domain (marked in an orange frame). Several interaction binding sites were located on synaptotagmin: tubulin (red stripped frame); calcium channels through syntaxin (gray stripped frame); and targeting signal to neurons that overlaps with the neurexin binding (blue stripped frame). Proteins (top to bottom): synaptotagmin, moth; CG3139 (iso A), beetle; synaptotagmin, mosquito; CG3139 (iso A), honeybee; CG3139 (iso C), fruit fly; CG3139 (iso A), fruit fly; CG3139 (iso A), fly obscura; synaptotagmin 1, human; synaptotagmin 1, opossum.

In an attempt to gain new information on the structure and function of presynaptic proteins, we applied a comparative view and conducted multiple sequence alignment (MSA) analysis of human and insects for representatives of the exocytotic machinery, VAMP-2, and synaptotagmin 1 (Figure 1). VAMP-2 is a short, evolutionary conserved protein of 120 to 220 amino acids with a SNARE-interacting domain and a single transmembrane domain (TMD) that crosses the synaptic vesicle membrane. Short signatures in VAMP's sequence that serve as recognition sites for tetanus and botulinum toxins [37] and the amino acids that are critical for VAMP targeting [38] are conserved from human to insects (Figure 1a). The sequence difference in the MSA is restricted to VAMP2 protein tails. A short proline-rich region that is responsible for VAMP2 interaction with synaptophysin [39] is not conserved. This is in accordance with the lack of synaptophysin in insect synaptic vesicles [40] (Table 1). On the other hand, a short region facing the synaptic vesicle lumen is highly conserved among all insects. Interestingly, there are two VAMP variants in honeybee that differ only in their luminal domain, enforcing a functional difference between these two variants (Figure 1). The possibility that a functional binding domain is located in the luminal domain is consistent with findings for other synaptic vesicle proteins, including synaptotagmin [41] and SV2 [42].

MSA of highly conserved sequences from human to insects was also performed for synaptotagmin (Figure 1b). Synaptotagmins belong to a large and diverse gene family that coordinate multiple signals with trafficking and with membrane fusion [5,43,44]. In the mammalian synapse, synaptotagmin 1 (and 2) is a genuine synaptic vesicle protein that serves as the calcium sensor and interacts with SNAREs as well as with the calcium channel [45]. In addition, synaptotagmin is a linker to the endocytotic adaptor protein AP2 [46]. The overall similarity of synaptotagmin between mammals and insects is high throughout the cytoplasmic region, but this similarity does not extend to the luminal region. In the cytoplasmic region, the domain that was postulated to interact with AP2 and with neurexin is strongly conserved, suggesting that not only is the main function of the protein conserved but also is its engagement in a rich protein interaction network.

Because endocytosis and membrane recycling are integral processes in presynaptic function, we compared stoned B (STNB) between human and insects [46] (Additional data file 4). Stoned genes (in insects StnA and StnB) are part of the protein lattice network that is involved in clathrin-mediated endocytosis at synapses. The conservation level of human stoned B (called SALF) is rather low (<50% sequence similarity). Several short signatures along the proteins act in the binding of AP2 subunits (for example, AP50 for StnB and α-adaptin for StnA). The number and the positions of these short signatures are not conserved in vertebrates and insects (Additional data file 4). In addition to the binding of AP2 by StnB, it binds to synaptotagmin 1 within the 300 amino acids in the carboxyl-terminal in the fly and human homologs. Stoned proteins may support synaptotagmin 1 recycling by mediating the association with the AP2 complex. Based on the MSA analysis, additional strongly conserved sequences are suggested (syntaxin; Additional data file 5). These sequences are probably essential in interactions between yet undefined partners that are common to mammals and insects. Most MSAs of PS120 show that the level of conservation is much higher among the insect sequences as compared with human or other organisms. We emphasize that MSA from insects to human for strongly conserved proteins (synaptotagmin, syntaxin 1A, and VAMP2) and for much less conserved genes (stoned B, SCAMP1, and synapsin 1) is instrumental in detecting overlooked sequences that may be important for protein interactions, protein modifications, and regulatory functions. The MSA for syntaxin 1 and synapsin 1 is included in Additional data file 5.

### Sequence conservation among the subunits of the exocyst complex

We tested whether the comparative genomics perspective is informative in studying the evolution of physical and functional complexes in exocytosis and trafficking. To this end, we tested the conservation levels for the various components of the exocyst.

**Figure 2:**
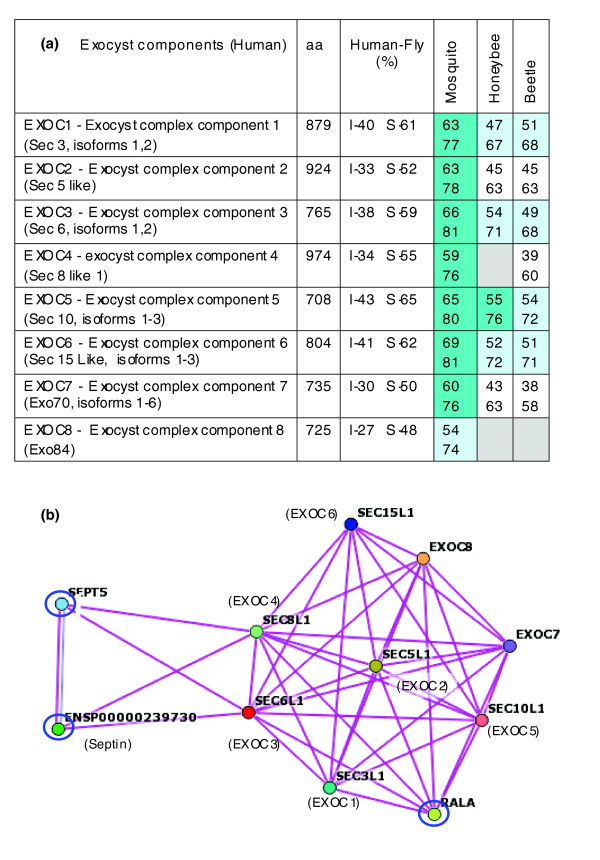
Exocyst protein interaction network in human and insects. **(a) **The subunits of mammalian exocyst (EXOC1 to EXOC8) and their yeast homologs (in parenthesis) are indicated. The percent of identity (I) and similarity (S) for human and the fly is shown. For mosquito, honeybee and beetle, the percentage identity and similarity (within each cell on top and bottom, respectively) relative to the *D. malenogaster *sequence are shown. Protein length is within 5% deviation between insect to their cognate human homolog. Dark blue background indicates similarity level above 75%, light blue indicates similarity above 65%, and white marks indicate similarity level below 64%. Gray background indicates that a homolog is missing. **(b) **Protein-protein interaction graph according to STRING tool (see Materials and methods). A tight interaction network extends from the exocyst to other partners (circled in blue) of small GTPase, RalA, and septin. aa, amino acids.

The exocyst is a large complex that was initially identified at the tip of the yeast bud. It participates in tethering vesicles to the plasma membrane. It coordinates exocytosis with small G-protein signalling molecules such as Ral-A, Arf6, and Rab11 [47]. The exocyst is composed of eight subunits that are denoted EXOC1 to EXOC8 (Figure 2a) and are homologs of the yeast Sec3, Sec5, Sec6, Sec8, Sec10, Sec15, Exo70, and Exo84 genes [48]. The level of conservation of the various subunits between human and fly range from 30% to 50% sequence identity (50% to 70% sequence similarity; Figure 2). The homologous relationship is evident and is supported by alignments that cover the entire protein length. However, among the insects, the mutual sequence conservation for EXOC8 is rather low (Figure 2a), because the honeybee and beetle homologs for EXOC8 are further diverged; hence, an apparent homology could not be assigned. Because the function of the exocyst relies on coordination of its subunits, we anticipated that EXOC8 would be missed during the task of genome annotation. This is further supported by the observation that several interacting proteins of the exocyst such as Ral-A [47] and septin 5 [49] are strongly conserved in all insects (Figure 2b). A search for sequence similarity in the honeybee and beetle genomes identified a supported mRNA for EXOC8 in honeybee and an apparently unprocessed sequence in the beetle genome (for details, see Additional data file 3). We conclude that physical complexes co-evolved because of similar evolutionary constraints.

### Evolutionary constrains on the subunits of the COP complex and the lysosome biogenesis complex

**Figure 3:**
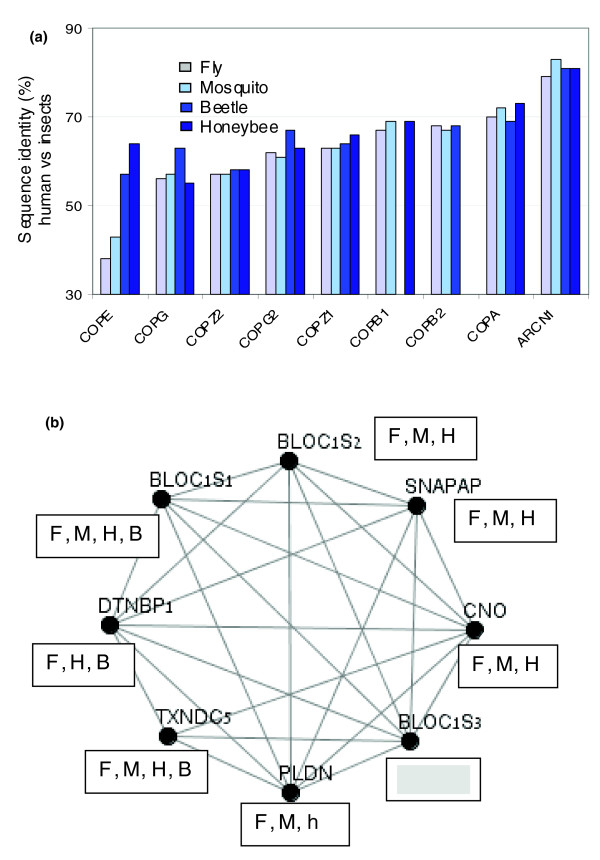
COP and BLOC interaction networks. **(a) **Sequence identity between human and insects of coatomer protein (COP)-1 proteins. The nine subunits of coatomer COP-1 are listed. The level of identity (%) between human and each of the four insect sequences is shown. The blue bars are color coded for the insect representatives as indicated. Note that for all proteins except CopE, the conservation level relative to the human ortholog is not different across the insects. Missing bars are due to missed annotations (as in Table 1 and Additional data file 3). **(b) **Biogenesis of lysosome-related organelles complex (BLOC)-1 in insects. The graph is based on confirmed interactions according to STRING scoring (see Materials and methods) for the eight subunits of mammalian BLOC-1. The identified homology to the fly (F), beetle (B), honeybee (H), and mosquito (M) are marked. Empty frame indicates no identified homologs in insects; small case letter indicates high sequence similarity that is only valid for a partial sequence. The interaction graph is based on identifying pair-wise interactions in BLOC-1. Information on individual subunits is available in Additional data file 2.

Coatomer protein (COP)-1 vesicles are principally involved in transport of cargo between the endoplasmic reticulum (ER) and early Golgi [50,51]. Specifically, they mediate both the anterograde flow of cargo through the Golgi to the cell surface and the retrograde retrieval of recycling proteins from late to early Golgi compartments. COP-1 is composed of 7 genes (α, β, β ', γ, δ, ε, and ζ subunits, additional genes resulting from duplication events, γ2 and ζ2) that are different in sequence and length. For example, whereas COPA (human homolog of α) is composed of 1,200 amino acid, COPZ (human homolog of ζ) consists of only 200 amino acids. Figure 3a shows the sequence identity of COP-1 components relative to human, for all four insect representatives. As may be observed, in eight of the nine genes the degree of conservation between human and insects varies little across insect species. An exception is COPE (εCOP-1), which, in addition to being the least conserved in the fly and mosquito, exhibits a large variation in the levels of conservation among insects. The honeybee COPE is significantly more conserved than that of the fly, mosquito or beetle homologs. We anticipate that COPE may display a different pace of evolutionary change that may be a result of its specific role in the COP-1 complex. Indeed, a role for this component in stabilizing rather than in the assembly of the COP-1 complex has been proposed [52].

The synapse is a compact structure with multiple organelles, including transport vesicles, early and late endosomes, lysosomes, and peroxisomes. Indeed, many of the PS120 representatives function in vesicle trafficking and sorting. Snapin (SNAPAP) is among the genes that are missing in some but not all insects. Snapin was initially identified as a SNAP-25 binding protein and a regulator of the interaction of synaptotagmin with the SNAREs [53]. The relevance of snapin in neurotransmitter release regulation was questioned [54], and instead it was postulated to be part of the biogenesis of lysosome-related organelles complex (BLOC) [55,56]. We compared the conservation of the subunits of BLOC-1 in human and insect (Figure 3b). The BLOC-1 complex is composed of eight short proteins (12 to 15 kDa) that are rich in helical structures. The composition of BLOCs is based on biochemical purifications and on localization studies [57], but the function of the individual subunits of BLOC-1 remains elusive.

A human homolog of snapin (SNAPAP; Table 1) is detected in honeybee, fly, and mosquito, but cannot be detected in the beetle genome (see Additional data files 2 and 3). BLOC1S2 and cappuccino are also missing in the beetle proteome, whereas BLOC1S3 is missing in all insects (Figure 3b). A detailed pair-wise interaction analysis showed that BLOC1S3 is peripheral and its interaction with other BLOC-1 subunits is only through BLOC1S2 [57]. Another component of BLOC-1 is dysbindin (DTNBP1). DTNBP1 is weakly conserved in insects (identity 26% to 28% from human to fly and beetle), and the fact that it is missing in both mosquitoes (*Anopheles *and *Aedes*) indicates that this is probably not due to annotation mistakes (Figure 3b). Interestingly, defects in DTNBP1 and other BLOC-1 components are linked to severe pathologies in humans [58]. Our findings are consistent with the notion that BLOC-1 is functional despite some missing components and suggest that there is some level of redundancy among BLOC-1 subunits in insects.

### Coordination in sequence conservation in biogenesis and trafficking protein complexes along the phylogenetic tree

**Figure 4:**
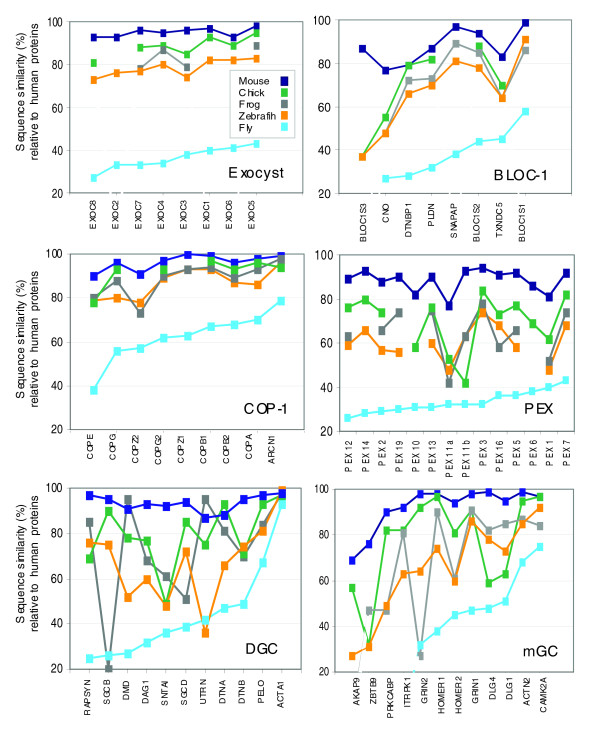
Evolution conservation among components of synaptic complexes. Conservation is measured by sequence identity (y-axis [%]) between human and five species: mouse (*Mus musculus*; dark blue), chicken (*Gallus gallus*; green) frog (*Xenopus laevis*; gray), zebrafish (*Danio rerio*; orange), and fly (*Drosophila melanogaster*; light blue). Data are sorted according to human-fly conservation. The conservation of each component in the complexes is shown. Shown are findings regarding the synaptic complexes that are associated with functional organization of the postsynaptic membrane: exocyst (EXOC; eight proteins; see Figure 2a), coatomer protein (COP)-1 (nine proteins; see Figure 3a), biogenesis of lysosome-related organelles complex (BLOC)-1 (eight proteins; Figure 2b); peroxisome biogenesis (PEX; 14 proteins); dystrophin glycoprotein complex (DGC; 11 proteins); and metabotropic glutamate receptor (mGC; 12 proteins). Note that the conservation range for fly proteins of the DGC and mGC spreads on a broad range, and for these complexes the conservation along the evolution tree is poorly coordinated.

The analysis of BLOC-1, COP-1, and the exocyst complexes (Figures 2 and 3) implies that the conservation levels for most subunits are similar within each complex and functional group. To test the generality of this observation along the evolutionary tree, we quantified the level of sequence identity in proteins that function in trafficking complexes and organelle biogenesis. The pair-wise sequence identity serves to reflect the conservation index. We tested the following organisms relative to human: mouse (*Mus musculus*), chicken (*Gallus gallus*) bony fish (Zebrafish; *Danio rerio*), frog (*Xenopus laevis*) and fly (*D. malanogaster*). Figure 4 shows the conservation relative to human proteins (measured as the percentage identity) for vesicle trafficking and organelle biogenesis complexes. We tested the presynaptic site protein complexes (exocyst and COP-1) and organelle biogenesis sets (BLOC-1 and peroxin biogenesis [PEX] genes, which participate in peroxisome biogenesis) and complexes from the postsynaptic site: the dystrophin glycoprotein complex (DGC), a complex that serves as a link between the cytoskeleton and the extracellular matrix in skeletal muscle cells [59]; and the active signaling complex of the metabotropic glutamate receptor (mGC), which includes glutamate receptors and their partners, such as cytoskeletal and post-translational modification enzymes.

In general, for the four sets of presynaptic sites, all tested species maintain a conservation index in a rather tight range, in which each complex exhibits a unique profile along the evolutionary tree. Specifically, the conservation of fly to human is in accordance with a high degree of coordination among these four complexes. The exocyst and COP-1 are the least diverged whereas the organelle biogenesis complexes (PEX and BLOC-1) exhibit a more active evolutionary divergence for at least some of their components (Figure 4). Although the components of COP-1 and BLOC-1 physically interact, the PEXs (peroxisome-related proteins) are a dynamic group of proteins with 14 gene products that function in executing the peroxisomal life cycle [60,61]. Each PEX protein is unique in length, structure, and function. The evolutionary conservation pattern is preserved across the five species included in this analysis, throughout the various components of the complexes. Presumably, the shared functions of the different components lead to their co-evolution.

To explore whether the coordination within complexes and functional groups along the evolutionary tree holds for other physical or functional complexes, we examined the DGC [59] and the active signaling complex of the mGC from the postsynaptic membrane [62]. Among the various proteins of these postsynaptic complexes, each species exhibits a different level of conservation relative to human. For example, DGC, the frog DMD (dystrophin), and UTRN (utrophin) [63] are almost identical to human, whereas SGCB and SGCD (β-sarcoglycan and δ-sarcoglycan) are poorly conserved. On the other hand, in zebrafish utrophin is poorly conserved whereas β-sarcoglycan and δ-sarcoglycan are more similar to human. A similar uncoordinated profile for conservation was shown for the mGC. We propose that for biogenesis, exocytosis, and trafficking complexes of the presynaptic sites (but not for postsynaptic signaling complexes), evolutionary constraints have led to co-evolution of the components.

### Presynaptic proteins participate in interconnected protein interaction graphs

**Figure 5:**
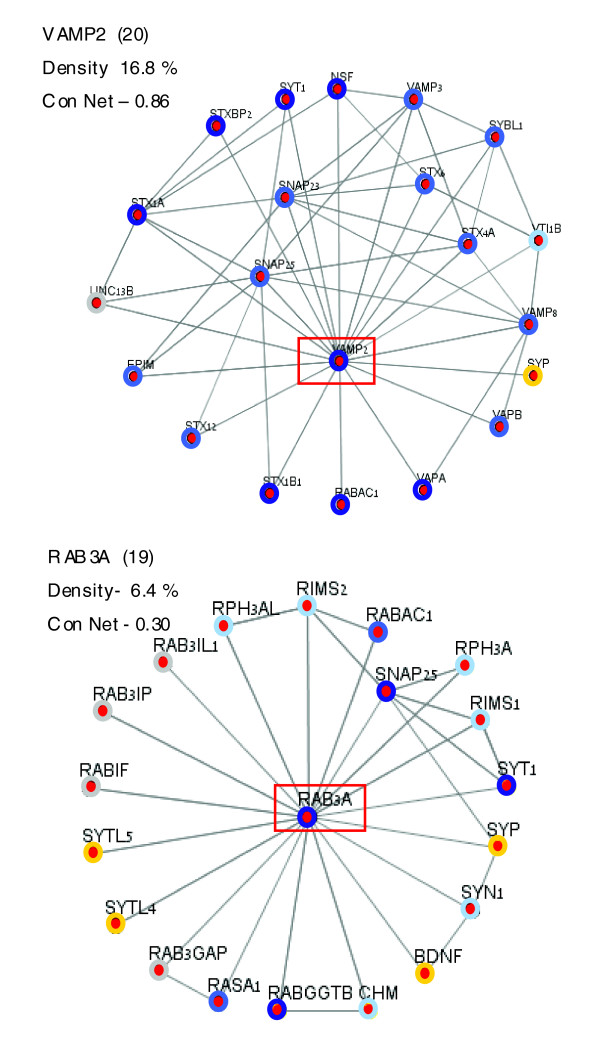
Interaction graphs of VAMP2 and RAB3A and their insects homologues. Human vesicle-associated membrane protein (VAMP)2 and RAB3A are shown as interacting graphs with their high confidence partners according to STRING tool (see Materials and methods). Proteins with close homologs in insects, sharing greater than 75% sequence similarity with a human homolog, are indicated by dark blue; proteins sharing 65% to 74% sequence similarity are indicated by light blue circles (Table 1). Proteins for which homologs are absent in insects are marked by yellow circles, and proteins whose sequences were absent because of missing annotations are marked with gray circles. For details, see Table 1 and Additional data files 2 and 3. Each of the central proteins (in red frame) is shown along its 'network conservation score' (Con Net), which measures the fraction of highly conserved proteins (>65% sequence similarity from insect to human) relative to all proteins in the graph. A quantitative measure for the density of protein-protein interactions in the graph is added (see Materials and methods, below). Note that the graph of VAMP2 is characterized by a high 'network conservation score' and larger 'interaction density' value relative to RAB3A.

Sequential protein interactions are fundamental to the lifecycle of the synaptic vesicle and to trafficking and organelle biogenesis in synapses. This leads to proteins that are engaged in multiple protein interactions. For example, more than 60 different interactions have been reported for syntaxin 1 and tens of interactions for synaptotagmin. Although some findings may result from spurious interactions, many have been experimentally confirmed and others are yet to be discovered. We illustrate this via VAMP2 as a prototype for an extremely conserved protein from human to insects. Figure 5 shows a graph centered on human VAMP2 along with 20 of its high-fidelity interacting proteins. Nineteen of these (excluding only synaptophysin) are conserved in all insects, and conservation for most of them (18/19) is very high (network conservation is 0.86). Another extreme case is that of the Rab3A protein (Figure 5b). Although the valence of Rab3A is very high (19), the properties of the two graphs are substantially different (Figure 5). Few Rab3A partners are missing in all insects and additional ones are missing in some insects, leading to a low conservation (network conservation 0.3).

The fraction of connecting edges in the graph relative to the maximal possible edges is a measure of the connectivity among interacting proteins. Density values for the interacting proteins of VAMP2 and RAB3A are 16.8% and 6.4%, respectively. Figure 5 illustrates proteins of the presynaptic apparatus that differ substantially in their valence, network conservation score, and density value.

**Figure 6:**
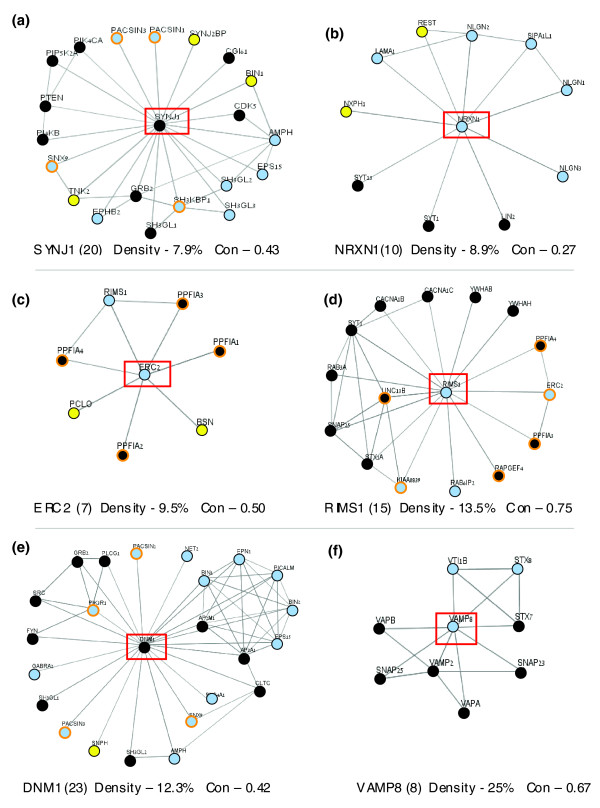
Presynaptic proteins participate in interconnected protein-protein graphs. The protein-interacting graphs are extracted from STRING tool and supported by the literature, experimental data, and strong homology inference. Only high confidence interactions are shown (see Materials and methods). The central protein in each graph (marked with a red frame) is a representative for **(a) **synaptojanin 1 (SYNJ1; a key signaling protein in endocytosis), **(b) **NRXN1 (in presynaptic membrane interaction and synaptogenesis), **(c) **ERC2 (an active zone organizer), **(d) **RIMS (a genuine component of the active zone), and **(e) **vehicle-associated membrane protein (VAMP)8 (a component of the exocytosis). Protein names are according to the official gene symbols (as in Table 1 and Additional data file 2). Protein valences (the number of direct edges from the vertex) are marked in parenthesis. Note that the graphs for RIMS, ERC2, and NRXN1 are of a relatively low connectivity. Protein vertices are colored according to the conservation index; proteins with a sequence similarity above 65% relative to a human homolog are framed in black, otherwise they are marked in light blue. Proteins that were missing in one or more of the insect representatives are framed in orange. Proteins that do not have insect homologs (as in Table 1 and Additional data file 2) are marked by a yellow circle. A quantitative measure for the density of protein-protein interactions in the graph is added as well as the network conservation score (Con).

We illustrate the properties of protein-protein interaction graphs for several representative proteins from the PS120 set (Figure 6; gene names are according to official symbols; see Additional data file 2). The protein interaction networks are supported by evidence from the literature, experimental data, and strong homology. Only high confidence interactions are shown (see Materials and methods, below). These proteins are as follows: VAMP8, a synaptic vesicle and exocytosis related protein; neurexin-1 (NRXN1), which acts in synaptogenesis and in the pre-post synaptic junctions; synaptojanin 1 (SYNJ1) and dynamin 1 (DNM1), which are endocytotic proteins that function in synaptic vesicle recovery and in clathrin-based endocytosis, respectively; and Rim-1 (RIMS) and Cast (ERC2), which are two active zone organizers.

The protein valence (defined as the number of direct edges from the vertex representing the protein) ranges from seven for ERC2 to 23 for DNM1. The graphs of RIMS, ERC2, and NRXN1 have relatively low connectivity. Specifically, in the NRXN1 graph there are only 14 edges, and for RIMS (with a valence of 15) just 29 connecting edges are observed. The density of the different graphs and their network conservation scores are marked (Figure 6). Note that for some of the interacting proteins no insect homologs are known (marked by a yellow circle; Figure 6). The low connectivity graphs are characteristic for additional proteins of the active zone and for some master regulators such as RAB3A (Figure 5) and LIN7A (Additional data file 6). DMN1, which is one of the central proteins of endocytosis, exhibits a mixed property in the protein interaction graph. Most edges are of low connectivity, but about one-third of the edges are highly connected. DMN1 and SYNJ1 valence is rather high (23 and 20, respectively) with only an intermediate network conservation score of 0.42 and 0.43, respectively. Note that for exocytosis proteins (namely VAMP2 and VAMP8), both the network conservation score and the density values are higher (Figures 5 and 6).

The interaction graphs for VAMP2 (Figure 5), VAMP8 (Figure 6), α-SNAP (SNAPA) and synaptotagmin 1 (SYT1; Additional data file 6), syntaxin 1 (STX1), and SNAP25 (not shown) are characterized by relatively high conservation and by high density values. The properties of the graphs for DNM1 and SYNJ1 are valid for numerous endocytotic proteins, including AP2A (Additional data file 6), clathrin, and amphiphysin (not shown).

### Valence of proteins in the interaction graphs and sequence conservation levels are positively correlated

**Figure 7:**
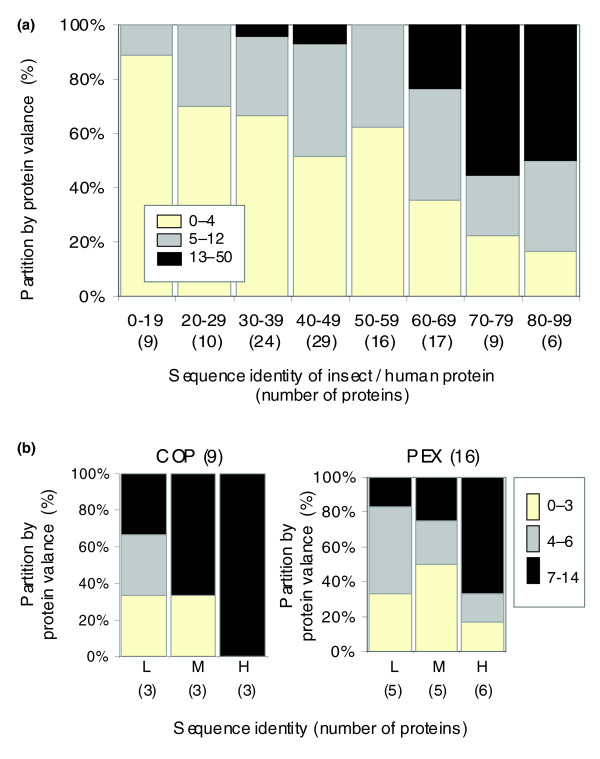
Valence of proteins in the interaction graphs and sequence conservation levels are positively correlated. **(a) **Genes from the presynaptic 120 genes (PS120) list were measured for their sequence conservation (percentage sequence identity between insects and human) and for the valance of each protein. The number of proteins for each sequence identity range is indicated in parenthesis. The number of protein partners for each PS120 is provided in Additional data file 5. Note that a positive correlation between the protein conservation index and the protein valance is evident only at highly conserved sequences (>50% identity). **(b) **Data from protein complexes (coatomer protein [COP]-1 and peroxin biogenesis [PEX]) follow a similar trend as the PS120. Proteins of each complex were divided into groups according to their sequence identity level (low [L], medium [M], and high [H]). The number of proteins in each group is indicated in parenthesis. PEX (16 proteins, including the 14 core PEX proteins; Figure 4) are weakly conserved between human and insects, while COP-1 (nine proteins; Figure 3a) exhibit a stronger conservation index. Note that the PEX and COP-1 proteins are not included in the PS120 set.

Almost all proteins in the PS120 gene list are engaged in multiple protein interactions (Additional data file 2). Interactions between proteins in the synapse occur throughout the processes of endocytosis, membrane fusion, protein recruitment, transport of vesicles, and organelle biogenesis. We tested whether conservation (as reflected by percentage sequence identity from insects to human) and the valence of all proteins are correlated. We considered only interactions with high confidence (see Materials and methods, below) and tested the two quantitative measures. Figure 7a shows the sequence similarity of all PS120 proteins as a function of the valence of human proteins. It is evident that the two quantities are positively correlated. Among the PS120 proteins, some exhibit extremely high numbers of interacting proteins (up to 50), as evident for signal transduction proteins, whereas others have no interacting partners. The latter could also be due to lack of current knowledge. Detailed information on PS120 protein conservation and valence is available in Additional data file 7. Note that correlation between conservation and protein valence is strongly associated with sequences that share greater than 50% identity (Figure 7). The positive correlation between conservation and protein valence is based on the full PS120 list. A similar analysis of components of functional complexes in trafficking (COP-1 and PEX; Figure 7b) is in accordance with the overall trend observed for PS120. (Note that these additional 25 proteins of COP-1 and PEX are not included in PS120.) We conclude that despite a relatively narrow range of sequence conservation among the components of each complex (Figures 2 and 3), a greater conservation score between insect and human is correlated with higher valence.

The next question that was addressed is whether the subsets of proteins that function primarily in endocytosis and in exocytosis exhibit different properties of sequence conservation and protein valence. To this end, we compiled two nonoverlapping subsets for endocytosis and exocytosis (24 proteins each; see Materials and methods, below). These two lists excluded proteins that are *bona fide *participants of both processes (for instance, DOC2, UNC13, PSCD1, and RIMS) or are connected to cytoskeleton modulation (such as ADD2, bassoon, CADPS, DLG1, KIF1A, and MYRIP). In the set of exocytotic and endocytotic proteins, the average protein valences are 10 and 7.9, respectively (*P *value for being identical = 0.423; Additional data file 8). However, for the exocytotic set the average valence for proteins with low conservation (<50% sequence identity) is 4.5, and 13.9 for those that share greater than 50% sequence identity. This result is significant (*P *= 0.034). In the endocytotic set, the valences of low and highly conserved proteins (<50% and >50% identity) were not significantly different (*P *= 0.605) and measured to be 7.2 and 8.6, respectively (Additional data file 7).

### Endocytotic proteins are long and composed of several repeated domains

**Figure 8:**
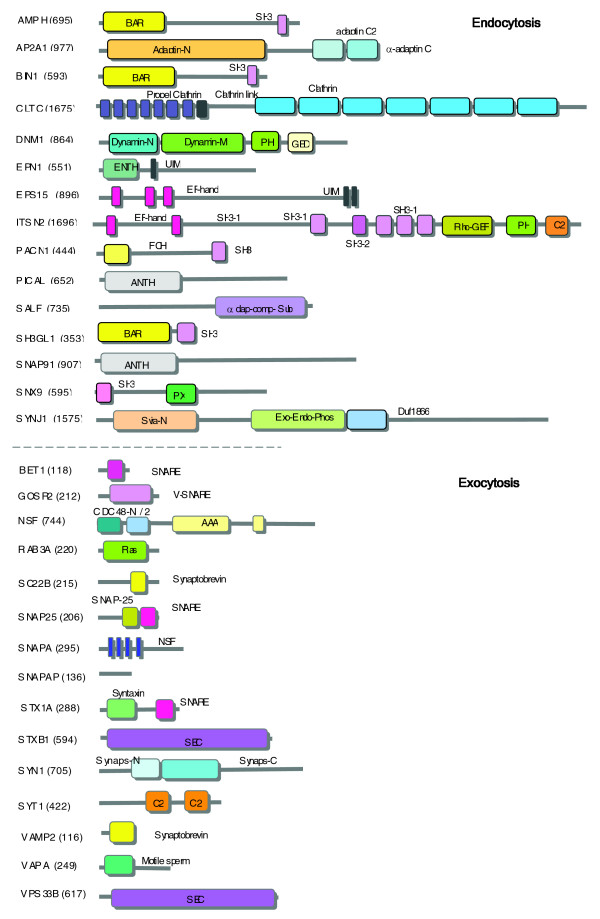
Exocytotic and endocytotic proteins exhibit different domain architectures. Molecular architecture of exocytotic and endocytotic proteins (15 representatives each; top and bottom frames, respectively). The complete lists and additional structural information is accessible in Additional data file 8. The proteins are drawn to scale, and the domain architectures are based on Pfam protein family. Domains are indicated by their colors. Detailed information on the properties of the domains is available in Additional data file 2 [part b].

We observed that the protein interaction graphs differ substantially with regard to their properties between essential proteins of the exocytotic and endocytotic sets (Additional data file 8). Specifically, several of the endocytotic proteins interact with proteins that are less conserved between insects and human (Figure 6). We tested the underlying molecular architecture of exocytotic and endocytotic proteins. Figure 8 shows a set of 15 proteins from each of the exocytotic and endocytotic sets. On average the endocytotic proteins are longer: 850 and 340 amino acids for the endocytotic and exocytotic proteins, respectively.

Additional features that were tested include the fraction of low complexity and coiled coil regions. It was shown that in both protein sets approximately 10% of the sequences are occupied by low complexity regions. However, coiled coil domains are detected mostly in endocytotic proteins. The most obvious difference is in the architecture of the exocytotic and endocytotic proteins, in that most endocytotic proteins are multi-domain proteins, some of them repeating several times within a protein and across the protein set. Examples are clathrin (CLTC; clathrin domains repeat seven times) and intersectin (ITSN2; with multiple SH3 domains). (For consistency, we applied the domain assignment according to Pfam [64].) Such features are rare among the exocytotic protein set (Figure 8). Some highly represented domains in the endocytotic protein set are short (Figure 8). These domains coordinate interactions with either short signatures (SH3), lipid moieties (PX and PH), or signaling ions (EF-hand). These are abundant domains that appear thousands of times in the animal kingdom and are used in a variety of cellular contexts, including outside the synapse.

The abundance of short domains with a broad specificity is rare in the exocytotic context. The domains marked as v-SNARE and t-SNARE, Synaptobrevin and Sec1 (Figure 8) are engaged in protein-protein interactions. Specificity is often achieved through elongated interfaces in helical regions [65]. An exception is the C2 domain, which appears in synaptotagmin. Although synaptotagmin is a genuine protein of exocytosis, it has some resemblance in its architecture to endocytotic proteins. Specifically, the C2 domain is broadly used in signaling proteins that are regulated by calcium in a context of membrane interactions. Many C2-containing proteins in the synapse are actually linkers of exocytosis and endocytosis [66], including BAIAP3, SYTL4, RIMS, and MUNC13 (Additional data file 2 [part b]). Roles of synaptotagmin and synaptotagmin-like proteins as linkers between exocytosis and endocytosis [46] and the cytoskeleton [67] have been proposed.

## Discussion

Comparative analysis of insect genomes focuses on evolutionary events on a scale of hundreds of millions of years. The numerous fly-related genomes (as in the case of *Drosophila pseudoobscura*) provide a snapshot of the evolutionary processes that occurred over tens of millions of years [3]. In this study we show that the rich source of data from insect proteomes is instrumental in deriving insights into the design principles of presynaptic function. It is demonstrated that comparative proteome analysis of insects to mammals provides new information on individual proteins (Figure 1); co-evolution of protein complexes that are involved in trafficking and organelle biogenesis (Figures 2 to 4), and the evolutionary constraints on proteins that are engaged in multiple interactions (Figures 5 to 7). This analysis takes advantage of the rich cellular and molecular data in the context of the CNS [68] from model organisms such as the worm, fly, and mouse [69,70].

### Membranous synaptic vesicle protein composition from human and insects is different

Mammalian synaptic vesicles were characterized by three major proteins [71]: SV2, synaptotagmin, and synaptophysin. Many of the synaptic vesicle membranous proteins appeared to be regulators of SNAREs and thus control neurotransmitter release. Examples are the transporter-like SV2, which controls synaptoagmin 1 [72], and synaptophysin, which controls VAMP-2 [73]. Based on a recent study on the composition of a generic mammalian synaptic vesicle [18], it was calculated that VAMP2 and synaptophysin are at a 2:1 molar ratio and both are the most abundant proteins of the synaptic vesicle membrane. In synaptophysin knockout mice, no changes in synapse function and brain morphology have been detected, but VAMP2 concentration on the synaptic vesicle membrane was shown to be markedly altered [74]. Along this line, the absence of synaptophysin gene in insects is intriguing (Table 1). Synaptophysin is part of a small family of four-transmembrane proteins that includs synaptogyrin, pantophysin, and synaptoporin, all of which are missing in insects. A genomic search revealed a weak similarity to synaptogyrin in honeybee supported by a transcript (XR_015081.1; hypothetical LOC552402 having mRNA of 704 nucleotides) and a weak homology in beetle for Synaptoporin (XM_967892.1; similar to synaptoporin, LOC661749). Despite its abundance in mammalian synaptic vesicles, Synaptophysin and the other members of the four-transmembrane protein set must be dispensable for the functionality of synaptic vesicles. This is in agreement with the findings of a recent study on *C. elegans *[75], in which complete removal of the synaptophysin gene family resulted in normal synaptic properties, synaptogenesis, and neuronal architecture. It is plausible that in insects VAMP accessibility is regulated by an alternative regulator. The extension of VAMP's luminal domain in insects is consistent with the possibility that such a domain is used for regulation (Figure 1). Candidates for insect synaptic vesicle proteins that face the lumen are synaptotagmin and SV2.

Synaptotagmin (Syt), the calcium sensor for neurotransmitter release in synapses, functions in the coupling of synaptic vesicle fusion to recycling. In mammals, synaptotagmin belongs to a large gene family of 16 genes. Among them, synaptotagmins 1 and 2 are exclusively functional in the synaptic vesicle lifecycle. In the fly genome, there are six representatives (close homologs of Syt1, Syt4, Syt5, Syt7, Syt9, and Syt12), four in mosquito (*A. aegypti *and *A. gambiae*), and four in the honeybee (close homologs of mammalian Syt1, Syt4, Syt5, and Syt7). Interestingly, several synaptotagmin-like proteins and alternatively spliced transcripts are detected in the fly. Among these are close homologs of Syt1 (CG3139, isoform D) and Syt7 (CG2381, isoform F); both variants lack the amino-terminal domain, including the transmembrane. These variants, although soluble, fully maintain their potential to bind endogenous synaptic regulators and to act as calcium sensors.

Although insects have only six genes, as opposed to 17 synaptotagmin genes in human, this is not the case for SV2, which is a transporter-like gene family of the synaptic vesicle membrane [76,77] that was implicated in modulating synaptotogmin [72]. The SV2 family is actually expanded in insects. At least three genes and three variants are evident in each of the insect representatives, suggesting the importance of multiple genes (Additional data file 9). Many expressed sequence tags and mRNA for SV2 homologs were detected from the fly head (not shown). This apparent high expression supports the notion that regulation of synaptotagmin might be executed by the rich collection of SV2 gene products. It is not known whether the different SV2 variants are all expressed in the same synaptic vesicle or whether they describe several subtypes. In general, the sequences of the variants and genes among insects are quite divergent. The expansion of the SV2 family in insects is shown in Additional data file 9.

Among the proteins that are missing in some of the tested insects is the Rab3 and its regulators. Rab function is controlled by a large group of regulators that affect interaction with the cytoskeleton, activation and inactivation of the GTP state, interaction with the membrane, and more [78]. For example, RAB3IL1, which is the GEF (guanine nucleotide exchange factor) for Rab3A, is missing in both flies and mosquitoes, but a close homolog is found in honeybee and beetle genomes. RAB3IL1 associates with inositol 6-phosphate kinase and provides another tier of Rab3 regulation in synaptic vesicle exocytosis. On the other hand, Rab3 GTPase-activating protein (Rab3GAP), which is responsible for switching between the active and inactive form [79,80], is missing from the honeybee genome. There is support for the indispensability of RAB3GAP in mammalian synapses in the literature; a mutation in the gene causes a severe brain developmental defect [81].

Surprisingly, many of the genes that are missing in one or more of the insects are linked to Rab3 regulation (Figure 5). We postulate that once some regulators are lost or impaired in their function, no positive selection is imposed on the other regulators. The result might be a rapid divergence beyond the level of detection by sequence similarity.

### Comparative genomics perspective on functional complexes

Comparative genomics approaches are powerful tools for gaining insights into evolution [82]. We suggest that these approaches be expanded to investigate functional groups and protein complexes (Figures 2 and 3). Some functional complexes bear more constraints than others (Figure 4). For example, in the fly exocyst components are within the 20% to 40% conservation index, and for COP-1 the range is from 40% to 80%. In these two complexes, the frog and zebrafish maintain about 80% conservation index throughout and thus are less informative. On the other hand, inspecting the conservation index of BLOCS1S3 shows that in all tested organisms (mouse, chicken, frog, zebrafish, and fly) it is the most diverged relative to the other components, and thus its specialized role in the BLOC-1 can be postulated.

We showed that within the COP-1 complex, only the ε-COP in insects exhibits a specialized evolutionary profile (Figure 3). This is in accordance with the observation from the mammalian COP-1 assembly [83]. It was demonstrated that ε-COP is the last subunit to be added during the assembly, and consequently the assembly of any other component is not dependent on its presence. Furthermore, in yeast ε-COP (called Sec28p) is shown to interact with α-COP genes. Unlike other COP genes, however, it is nonessential for cell viability. It was proposed that ε-COP is not necessary for the *in vivo *assembly of COP-1 coatomer but for stabilizing α-COP [52].

The co-evolution in components of the same complex is not a general trend in the synapse. The DGC is part of the overall architecture of the nerve-muscle postsynaptic site. Indeed, the DGC in the skeletal muscle membranes shows that the components evolve in an uncoordinated mode across individual species. More than ten proteins strongly interact to form the DGC functional complex. The importance of this complex in mammals is evident because mutations in many of the genes account for the pathologies of muscular dystrophies [59,84]. There are numerous complexes of the postsynaptic sites that in the CNS are localized to the PSD. These complexes show a similar trend to DGC, including mGC (Figure 4), the NMDA-MaGuk-associated (NRC/MASC) and the AMPA receptor (ARC) complexes [62] (not shown). We attribute this to the functional composition of the PSD complexes. The individual components are a combination of cytoskeleton, scaffolding, signaling enzymes, receptors, channels, and adaptor proteins. The assembly of such complexes is a result of the multiple PDZ and a few adaptors [85]. We attribute the evolutionary differences in coordination (Figure 4) in the presynaptic and PSD complexes to the fact that the latter include a plethora of components, many of which are not exclusive to the synapse. We argue that a careful comparative analysis of functional complexes identified distinctive design principles for the complex in trafficking and biogenesis versus postsynaptic complexes. The architectural properties of the PSD resemble adhesion molecules and their evolutionary constraints [86].

### Architectural design principles in exocytosis and endocytosis

The PS120 represents the core of the presynaptic gene list. The two subsets could (somewhat artificially) be divided into the exocytotic and endocytotic proteins. It is clear that the two processes are tightly connected in time and space [23,87]. Nevertheless, fundamental differences could be assigned to each of the subsets in terms of protein length, conservation level and protein valence, abundance of coiled coil regions, membranous interactions, and so on. We argue that most of these features underlie the design principles for the functional distinction between these two processes.

Based on protein-protein interaction graphs, a positive correlation between the presynaptic protein conservation score and their valence was demonstrated (Figure 7). Although the average protein valence is high (7.9 to 10 interacting proteins on average), it is not significantly different for the set of exocytotic and endocytotic proteins. The trend seen in Figure 7 is reflected mostly by the exocytotic but not the endocytotic proteins (Additional data files 7 and 8).

In the animal kingdom, the exocytic event is rapid (ranging from milliseconds to seconds), accurate, and triggered by a combination of depolarization and calcium signals that lead to synaptic vesicle fusion. On the other hand, the endocytotic machinery is slower and more diverged. For example, after a massive synaptic vesicle exocytosis from the Torpedo electric organ, the synaptic vesicles are recovered within 18 hours [88], and after repeated stimuli in the mouse brain the replenishment of synaptic vesicle pools is completed within 10 to 20 minutes and the recovery of a single synaptic vesicle is over within a few seconds [89]. We suggest that the modes for synaptic vesicle recovery are subject to large variations, and in evolutionary terms the high connectivity for the involved proteins has remained despite substantial sequence divergence. This observation is not restricted to insect endocytotic proteins. A recent study on the properties of endocytotic proteins [90] indicated that the high connectivity in protein-protein interaction reflects the spatial and temporal constraints of the process. Furthermore, endocytosis is not restricted to presynaptic function and the proteins often act in many cellular contexts, often by interacting with nonsynaptic protein partners.

Many of the endocytotic proteins have pleiotropic functions that are of varying importance in different species. This property differs from most exocytotic proteins. For example, in the mouse, amphiphysin (AMPH; Figure 8) regulates but it is not essential for synaptic vesicle recycling. In the fly, amphiphysin is responsible for the organization and structure of the muscle postsynaptic membrane and is not involved in synaptic vesicle recycling [91]. Thus, even amphiphysin, which is one of the hallmarks of the synaptic vesicle recycling apparatus, appears to be replaceable and prone to rapid changes in its sequence. We assume that this pleiotropicity functions in the endocytotic set of proteins (also applied to PSD proteins and adhesion proteins; not shown), underlying their relatively rapid evolution.

In addition to the protein valence and conservation argument, the protein structure and domain composition of endocytotic and exocytotic proteins are substantially different (Figure 8). What are the principles that led to the substantial difference in the exocytotic and endocytotic proteins that is maintained throughout the evolutionary tree? The abundance of repeated domains in endocytotic proteins (Figure 8) underlies the need to recruit multiple partners in parallel and to create a physical mesh of proteins. A partition of endocytotic proteins by the properties of their interaction graphs was presented [90]. Because numerous proteins and interacting domains were structurally resolved within the context of exocytotic [92] and endocytotic proteins, we currently seek a framework that combines analytical measurements of synaptic protein evolution and their structural features.

On a functional rather than structural level, the processes of endocytosis and exocytosis rely on multiple preparatory steps [23]. We attribute the difference between the processes mainly to these steps. In exocytosis, a sequential exchange of protein-protein interactions leads to priming and SNARE complex formation [19]. During the preparatory steps for endocytosis, an intimate interaction with the lipid properties must occur. The properties that are needed for recognizing lipids dominate the domain architecture of most endocytotic proteins. These proteins include the adaptors, sensors for phosphatidylinositol 4,5-bisphosphate, domains that induce membrane curvature and others (for details, see Additional data file 2). Among the domains that are enriched in endocytotic proteins (Figure 8 and Additional data file 8) are PX and PH, which bind phosphoinositides with different specificity; SH3, which binds to a short proline-rich signature; ENTH (epsin amino-terminal homology) domain, which forms a binding pocket for inositol triphosphate ligand; and BAR (Bin-Amphiphysin-Rvs) domain, which participates in membrane curvature; among others. Most exocytotic domains such as v-SNARE and t-SNARE still bind to different partners, but the binding is different in that it is sequential and exclusive, and it covers a large extended protein-protein interface segment [65]. An extreme example is the four-helix buddle SNARE complex [93].

Many of the exocytotic proteins but not the endocytotic proteins contain one or more TMDs (Additional data file 8). This leads to two important outcomes for the exocytotic proteins: the location of membranous proteins to the appropriate site in the synapse is already dictated through the secretory pathway by the sorting machinery; and most of these proteins carry post-translational modifications that were acquired during their maturation in the endoplasmic reticulum and Golgi. Exceptions are proteins such as Rab3A and SNAP-25 that, although lacking a TMD, are modified to ensure their covalent membrane association. Clearly, in cases in which a modification is associated with a protein function, this leads to additional evolutionary constraints on the modification sites. For example, synaptotagmin undergoes both N-glycosylation and O-glycosylation. The N-glycosylation was shown to be essential for redirecting synaptotagmin to the synaptic vesicle membranes [94], whereas the O-glycosylation is enhanced and triggered by VAMP-dependent interaction. Indeed, for synaptotagmin both sites are fully conserved along a broad evolutionary distance [67]. At present, experimental data on the functional importance of conservation of post-translational modifications from insect to human and along the evolutionary tree are lacking for large number of presynaptic proteins.

Several endocytotic proteins (but notably not the exocytotic proteins) include regions that are defined as coiled-coil domains (Additional data file 2 [part b]). Surprisingly, many of the architectural principles of endocytotic proteins are shared with the complexes of the PSD. This unified principle ensures multiple and rich connections to the cytoskeleton (in the case of PSD) and the lipids (in the case of endocytosis). As a result of their structural architecture, the PSD core proteins are subjected to rapid divergence, leading to a faded signal in the sequences from human to insects. We propose that this same principle is valid for the lack of bassoon and piccolo in insects genomes.

## Conclusion

Analysis of key presynaptic proteins from numerous insect proteomes yields insights into evolutionary processes that occurred more than 350 million years ago. We demonstrated how an approach based on comparative genomics reveals erroneous and missing annotations. This methodology also reveals instances of gene gain and loss in insect genomes. We conclude that strong evolutionary constraints have led to the co-evolution of protein complexes that are involved in trafficking and organelle biogenesis. Furthermore, presynaptic proteins that participate in multiple protein interactions in exocytosis exhibit high sequence conservation. The analogous statement does not apply, however, to endocytosis. Finally, we discuss the relationship between connectivity and sequence conservation in these two protein sets. We explain these differences in terms of the particular architectural design of these proteins, which is adapted for specific phases in the synaptic vesicle's lifecycle.

## Materials and methods

### Compiling the presynaptic PS120 collection

The list of proteins known as PS120 is a compilation of human genes that are related to the function of the synaptic vesicle life cycle. The proteins are indexed by their human official gene symbol. The conversion of the official gene symbol to protein accessions (from UniProt or National Center for Biotechnology Information [NCBI] protein collection) is performed using Protein Information Resource retrieval system [95]. This protein list provides a partial but overlapping union of collections from several resources: Mass spectrometry (MS) proteomics analysis from biochemical purified synaptic vesicles [18]; biochemical and genetic studies and literature [9,96]; SynDB collection, which includes synaptic process genes and their orthologs in multiple species, including human and fruit fly [97]; GO assignments [26] for the terms 'asymmetric synapse', 'exocytosis', 'synaptic vesicle', 'endocytosis', 'pre-synapse'; and the presynaptic gene index of mammalian genes [27]. The list was compiled manually to avoid redundancy.

The PS120 is an apparently complete collection of representatives of proteins that participate in vesicle trafficking, synaptic vesicle lifecycle, and presynaptic organization. Note that the 150 set of presynaptic genes accounts for 45 genes that are included in our PS120, but the additional 75 proteins were not represented in the report by Hadley and coworkers [27]. For example, there the 17 synaptotagmins and 15 syntaxins [27] that are represented by three synaptotagmins (synaptotagmin 1, 5, and 9) and one syntaxin (syntaxin 1A) in the PS120. From the approximately 60 Rab proteins known in human, only a small number of representatives that are directly involved in synaptic vesicle function is listed (Rab27 and Rab3). In addition, protein variants are not listed; for instance, only Rab3A (but not Rab3B, Rab3C, and Rab3D) is listed. We excluded proteins that function in signal transduction, including kinases and phosphatases. Specifically, the ten calcium calmodulin protein kinases that are included in the 150 gene collection [27] are excluded. We also excluded proteins that function in cell adhesion [98,99]; synapse maintenance and development, including growth factors and extracellular matrix; presynaptic channels and G-protein-coupled receptors [100]; and neurotransmitter transporters and multiple uptake mechanisms [101].

### Identifying orthologs in insects

Orthologs for each of the human PS120 list with insects were defined by the top reciprocal hit using BLAST2 [102]. BLAST searches were restricted to the nonredundant insect database from NCBI [103] and to the UniProt protein database [104]. The insect representatives that were used throughout are the fruit fly *D. melanogaster*, the honey bee (*A. mellifera*), red flour beetle (*T. castaneum*), and one of the two mosquitoes (*A. gambiae and A. aegypti*). The identification was compared with the preprocessed list of Ensembl database [105]. Detecting homologs in a genomic scale followed the procedure described in the ProtoBee database [106], in which annotations are assigned through hierarchical classification. Sporadic poor alignments and large deviation in protein length were used as evidence of false homology assignments. In instances in which an orthologous relation could not be validated, a direct search in Genome Sequences Centers and in specialized insect database was performed. The tBLASTn [103] was applied using a protein sequence against the translated nucleotide database in all six frames. Top hits were manually tested to separate spurious similarity for missed annotated genes. We applied the following to detect remote homologs and to resolve borderline similarity level: VectorBase, which focuses on *A. gambiae*, *A. aegypti*, *Ixodes scapularis*, *Culex pipiens*, and *Pediculus humanus *[107]; FlyBase, which focuses on a comparative view for a dozen *Drosophila *species. [108]; Baylor Genome Center (for Honeybee and Beetle genomes) and other genome centers (see list in Additional data file 1); and position-specific iterative BLAST [102].

The percentage identity and similarity were used as comparative measures. Conservation score was determined based on the fraction of proteins in the graph with at least 65% sequence identity between a human protein and its closest insect protein, and the total numbers of proteins in the graph. BLAST identity and similarity levels were recorded and both measures are strongly correlated (R^2 ^= 0.9315). For simplicity we used the identity measure (expressed as a percentage) unless stated otherwise. ClustalW [109] was used to produce MSAs. ClustalW MSA was used for neighbor-joining phylogeny reconstruction.

### Protein-protein interaction graphs

Networks of protein-protein interactions are based on the STRING web tool [110]. The database of STRING unifies most types of protein-protein associations, including direct and indirect associations. Most high quality experimental data covers model organisms. In some instances inference of known interactions from model organisms to other species based on orthology of the respective protein was considered. The numerical confidence score used was >0.9. The score was based on homology, experimental data, and text mining. The high confidence (>0.9) ensured that many of the interacting partners were supported by independent evidence types. We used a threshold for up to 50 interacting proteins and a score of >0.9 throughout. The maximal number of interacting was limited to 50 (only for calmodulin and 14-3-3 was this number below the reported interactions).

Density value was calculated from the ratio of the high-quality edges in a graph (following removal of the central protein and its direct edges) and the maximal edges possible. In a graph with *n *vertices, the maximal number of edges is *n *(*n *- 1)/2.

### Functional assignments

The nonoverlapping sets of endocytosis and endocytosis sets (24 proteins each) are based on GO annotations [26] and assignments in SynDB [97]. Because coverage of GO annotations for the PS120 is limited, we added proteins from the manual assignment of SynDB [97]. The endocytotic set is mostly based on 'endocytosis' and 'recycling', and for the exocytotic set we merged 'trafficking' and 'exocytosis'. We deleted proteins that are genuine linkers of the two processes from these lists.

Statistical significance (*P *value) of the properties of nonoverlapping sets of endocytosis and endocytosis sets was calculated by a standard Student's *t*-test, with the null hypothesis that the means of two independent sets are equal.

### Sequence features and domains

Pfam (version 22, 9300 protein families) [64] was used for domain assignment. TMHMM2 was used to predict the location and topology of transmembrane helices [111]. Coiled coils are detected using the method of COILS [112]. Low complexity regions are predicted using the SEG program and activated by the ProteinPredict meta-server [113].

## Abbreviations

BAR (Bin-Amphiphysin-Rvs); BLOC, biogenesis of lysosome-related organelles complex; CNS, central nervous system; COP, coatomer protein; DGC, dystrophin glycoprotein complex; GO, Gene Ontology; mGC, metabotropic glutamate receptor; MSA, multiple alignment sequence; PEX, peroxin biogenesis; PS120, presynaptic 120 genes; PSD, postsynaptic density; SNAP, Soluble NSF attachment protein; SNARE, SNAP-receptor; SV, synaptic vesicle; TMD, transmembrane domain; t-SNARE, target membrane SNARE; v-SNARE, vesicle SNARE; VAMP, vesicle-associated membrane protein.

## Authors' contributions

CY and NM contributed equality to the data acquisition, analysis, and interpretation of data. ML contributed to the design of the analyses and to the drafting of the manuscript. All authors have read and approved the manuscript.

## Additional data files

The following additional data are available with the online version of this paper. Additional data file 1 lists the proteomes from multiple insect species and their evolutionary relatedness. Additional data file 2 lists the set of 120 presynaptic protein prototypes in human relative to their insect homologs. Part a provides a detailed information on gene names, synonyms, major partner proteins, and conservation levels from human to insect homologs. Part b is a complementary table based on the UniProtKB database [104].

Additional data file 3 lists the 44 genes that had no clear homologs in certain insects. Additional data file 4 is a ClustalW based MSA of stoned B and SCAMP. Additional data file 5 is a ClustalW-based MSA of the SNARE syntaxin 1 and synapsin. Additional data file 6 shows representative graphs of interactions for six proteins, based on the STRING tool [110]. Additional data file 7 provides a detailed table of the PS120 gene set, including the level of sequence identity and similarity (as a percentage between human and insects), and the protein valence according to the STRING tool. Additional data file 8 presents a detailed table of gene functions in endocytosis and exocytosis (24 genes each). Additional data file 9 is the ClustalW-based MSA of the family of SV2 in insects and a tree-like representation based on distances from the MSA.

## Supplementary Material

Additional data file 1 Provided is a summary that lists the proteomes from multiple insect species and their evolutionary relatedness. A list of insect genome centers is compiled from NCBI Genome resources [114]. Click here for file

Additional data file 2 Part a provides a detailed table of the PS120 gene set and associated related information on gene names, synonyms, major partner proteins, and conservation levels from human to insect homologs. Part b is a complementary table based on the UniProtKB database [104]. Click here for file

Additional data file 3 Listed are the 44 genes that had no clear homologs in certain insects. After homology search in the nucleotide databases, the majority could be accounted for as missed annotated genes. Click here for file

Additional data file 4 Provided is a ClustalW-based MSA of stoned B and SCAMP. Click here for file

Additional data file 5 Provided is a ClustalW-based MSA of the SNARE syntaxin 1 and synapsin. Click here for file

Additional data file 6 Provided are representative graphs of interactions for six proteins based on the STRING tool [110]. Click here for file

Additional data file 7 Provided is a detailed table of the PS120 gene set with the level of sequence identity and similarity (expressed as percentage between human and insects) and the protein valence according to the STRING tool. Click here for file

Additional data file 8 Presented is a detailed table of gene functions in endocytosis and exocytosis (24 genes each). Information on the number of interacting proteins, level of sequence identity between human and insect, and additional structural properties is included. Click here for file

Additional data file 9 Presented is the ClustalW-based MSA of the family of SV2 in insects and a tree-like representation based on distances from the MSA. Click here for file
