# Supplementary material for: Evolution of insect proteomes: insights into synapse organization and synaptic vesicle life cycle
Source: Genome Biol. 2008 Feb 7;9(2):R27. doi: 10.1186/gb-2008-9-2-r27 (PMC2374702; doi:10.1186/gb-2008-9-2-r27)
Supplement: Additional data file 2 — Part a provides a detailed table of the PS120 gene set and associated related information on gene names, synonyms, major partner proteins, and conservation levels from human to insect homologs. Part b is a complementary table based on the UniProtKB database [104]. [file gb-2008-9-2-r27-S2.doc]

**Additional data file 2A**

|  | **PROTEIN**  **FAMILY** | **GENE**  **SYMBOL** | **ALTERNATIVE NAME** | **SIM**  **(mis)** | **MAJOR PARTNERS** | **CELL** | **PROC.** | **HUMAN - INSECTS** |
| --- | --- | --- | --- | --- | --- | --- | --- | --- |
|  | β-adducin | ADD2 | similar to hu li tai shao (insect) | C | RPH3A | Cyto |  | I -35%, S- 56%, aa: H-I 720 |
|  | Amphiphysin | AMPH | Isoform 1 of Amphiphysin | C | SYNJ1, DNM1 |  | Endo | I-33%, S-53%, aa H-700, T-450 |
|  | α-Adaptin | AP2A1 | AP-2 complex subunit alpha-1 | A | EPS15, DNM1 |  | Endo | I-71%, S-82%, aa: H-I:940 |
|  | AP-3 | AP3D1 | Adaptor related protein complex 3 (neuronal) μ1, σ, β, δ | A | LAMP1 |  | Endo | I-70%, S-86%, aa: H-I:420 |
|  | Mint | APBA1 | Munc18-interacting protein 1 | C | STXBP1 |  |  | I-49%, S-63%, aa: H-840, T-1100  *Only partial and weak in Honeybee* |
|  | Adapter protein X-11 β | APBA2 | Amyloid β A4 precursor protein-binding family A member 2 | B ( *) | LIN2, LIN7A | Cyto |  | I-60%, S-74%, aa: H-750, D-1170  *Not in Beetle, Many variants in fly* |
|  | ADP-ribosylation factor | ARF1 | ADP-ribosylation factor 1 | A | PSCD1, GGA1, COPB |  | Endo | I-95%, S-99%, aa: H-I:180 |
|  | ADP-ribosylation factor | ARF6 | ADP-ribosylation factor 6 | A | PSCD2, RALA |  | Endo | I-97%, S-100%, aa:H-I:180 |
|  | ARF - GEF | ARFGEF2 | Brefeldin A-inhibited guanine nucleotide-exchange protein 2 | B (*) | ARF1, EXOC7 |  | Endo | I-61%, S-74%, aa:H-1790, T-1660  *Not in Honeybee* |
|  | Arfaptin | ARFIP2 | ADP-ribosylation factor (ARF) interacting protein 2 | B | ARF6 |  |  | I-63%, S-80%, aa: H-I:340 |
|  | Vesicular V0 | ATP6V0C | Vacuolar ATP synthase 16 kDa proteolipid subunit | B | ATP6V | M | Exo | I-82%, S-89%, aa: H-I:160 |
|  | BAI1-associated protein | BAIAP3 | Isoform 1 of BAI1-associated protein 3 | C (*) | BAI1 |  | Exo-Endo | I-33%, S-49%, aa:H-1190, T-1260  *Not in Honeybee* |
|  | Bet 1 homolog | BET1 |  | C | NSF, GOSR1, SYX5 | M | Exo | I-52%, S-62%, aa: H-I:120 |
|  | Bridging integrator | BIN1 | ‎Myc box-dependent-interacting protein 1 | C (*) | ESP15, SYNJ1, RIN2 |  | Endo | I-36% , S-53% aa: H-I:450  *Bin3 is missing in Insects* |
|  | BLOC1 | BLOC1S1 | Biogenesis of lysosome-related organelles complex 1 | A (*) | CNO, PLDN, MUTED |  |  | I -65%, S- 81%, aa: H-I:130  *BLOC1S2 not in Beetle* |
|  | Bassoon | BSN | Aczonin | C (*) | RIMS, UNC13 | Cyto |  | *Not in insects.* H-3930 |
|  | CaV2.1 | CACNA1A | P/Q type Voltage dependent Ca^2+^ channel | B | SYT1, SYX, SNAP25, Gα,βγ | M | Exo | I-57%, S-73%, aa: H-2500, D-1850 |
|  | Caps | CADPS | Ca^2+^ dependent secretion activator | B (*) | D2 receptor |  |  | I-54%, S-69%, aa: H-1230, T-1440  *Not in Mosquito* |
|  | Calmodulin | CALM2 | Phosphorylase kinase, delta | A | [CACNA1C](http://string.embl.de/newstring_cgi/display_single_node.pl?taskId=Tc1ON8eyd6Bx&node=311599&targetmode=proteins), [ITPKA](http://string.embl.de/newstring_cgi/display_single_node.pl?taskId=Tc1ON8eyd6Bx&node=310015&targetmode=proteins), CASKIN | ST |  | I -97%, S- 99%, aa: H-I: 150 |
|  | Lin-2 homolog | CASK / LIN2 | Calcium/calmodulin-dependent serine protein kinase | A | NRXN1, RAB3A, APBA1 | EZ |  | I -67%, S- 82%, aa: H-I: 920 |
|  | Clathrin heavy chain | CLTC | CLTC Isoform 1 of Clathrin heavy chain 1 | A | ESP15, SYNJ1, DNM1, AP2 |  | Endo | I-81%, S-90%, aa:H-I:1680 |
|  | Cappuccino | CNO | Cappuccino homolog | C (*) | BLOC-1, SNAPAP, PLDN |  |  | I -31%, S- 51%, aa: H-220, A-170  *Not in Beetle, weakly conserved* |
|  | Caspr1 | CNTNAP1 | Neurexin 4, contactin associated protein 1 | C | KCNA2, SRC |  |  | I- 29%, S-47%, aa: H-1380, A-1290 |
|  | Synaphin | CPLX2 | Complexin 2 | C (*) | SNARE, SNAP |  |  | I -41%, S- 58%, aa: H-I: 130  *Many isoforms in Fly. CPLX4 not in Honeybee, Mosquito & Beetle* |
|  | SAP97 | DLG1 | Synapse-associated protein 97 | B | LIN2, GRIN2A | Cyto |  | I- 59%, S-74%, aa: H-850, D-970  *Weakly conserved, Many isoforms in Fly* |
|  | Cysteine string protein | DNAJC5 | DNAJ (HSP40) homologue | B | HSP, CACNA |  | Exo | I-59%, S-74%, aa: H-200, T-240 |
|  | Dynamin | DNM1 | Dynamin 1 | A | AP2, AMPH |  | Endo | I-73%, S-89%, aa: H-I:860 |
|  | Doc2 | DOC2B | Double C2-like domains, β | C | UNC13, STXBP1 |  | Exo | I-43%, S-60%, aa: H-400, T-550  *Weakly conserved* |
|  | Testilin | EHD1 | EH domain-containing protein 1 | A | SNAP29, EHBP1 |  | Endo | I -67%, S- 82%, aa: H-I: 530 |
|  | Epsin | EPN1 | Isoform 1 of Epsin-1 | A |  |  | Endo | I-43%, S-56%, aa:H-I:550 |
|  | Eps15 | EPS15 | Epidermal growth factor receptor pathway substrate 15 | C | AP2, DNM1, SYNJ1 |  | Endo | I-32%, S-46%, aa: H-740, T-930  *Weakly conserved* |
|  | RAB6-interacting CAST family | ERC1 | Isoform 1 of ELKS  RAB6-interacting CAST family | A | RAB6, RIMS1 |  |  | I -37%, S- 52%, aa: H-1800, T-1370  2 paralogs in insects |
|  | Exocyst | EXOC6 | Exocyst complex component 6 | B (*) | EXOC complex |  |  | I-51%, S-70%, aa: H-800, A-760  *Some EXOC missing in Beetle* |
|  | Exophilin | EXPH5 | Slp homolog lacking C2 domains | C (*) | RAB27A |  | Exo | I-21%, S-40%, aa: H-1990, A-880  *No homologue in Insects* |
|  | Syntabulin | FLJ20366 ‎ | Isoform 1 of Syntabulin -Golsyn | C (*) | STX1A, KIF5B | Cyto |  | aa: H-660  *No homologue in Insects* |
|  | Neuromodulin | GAP43 | Growth associated protein 43 | B (*) | S100B, CALM1 | Cyto |  | aa: H-240  *No homologue in Insects* |
|  | Rab GDI | GDI2 | Rab GDP dissociation inhibitor 2 | A | RAB |  |  | I-66%, S-82%, aa: H-I:450 |
|  | P-selectin | GMRP | GMP140, SELP | C | STX17 |  | Exo | I-25%, S-38%, aa: H-770, A-1130 |
|  | CFTR-associated ligand | GOPC | Golgi-associated PDZ and coiled-coil motif | B (*) | STX6, CFTR |  |  | I-48%, S-66%, aa: H-460, T-790  *Not in Fly, Honeybee and Mosquito* |
|  | Membrin | GOSR2 | 27 kDa Golgi SNARE protein, member 2 | B | NSF, SNAPA |  | Exo | I-48%, S-69%, aa: H-I: 210 |
|  | Hepatocyte GF regulated tyrosine kinase substrate | HGS |  | C | SNAP25, SNX1 |  |  | I-45%, S-56%, aa: H-790, A-830 |
|  | Intersectin | ITSN2 | SH3 domain-containing protein 1B | C | EPS15, SYNJ1 |  | Endo | I-33%, S-50%, aa: H-1700, D-1580 |
|  | Kinesin family member | KIF1A | Kinesin family member 1 | B | TUBB |  |  | I-59%, S-73%, aa: H-I: 660 |
|  | Lamp | LAMP1 | lysosomal-associated membrane protein 1 | C | AP1, AP3 |  | Endo | I-26%, S-44%, aa: H-420, A-320 |
|  | Lin7 homolog | LIN7A | Lin7 homolog A, MALS-1 | A | SAP97, MINT, NRXN1 | Cyto |  | I-79%, S-90%, aa: H-230, T-200 |
|  | CIRL | LPHN1 | α-Latrotoxin receptor, isoform 1 | C (*) | SYT3, Shank |  | Exo | I-38%, S-55%, aa:H-1480, T-1320  *Not in Beetle and Mosquito, Weak conservation* |
|  | Ras specific guanine releasing factor | MSS4 | Rabif, Mammalian suppressor of sec4 | C (*) | RAB, PRH3A,  PIP5K |  | Exo | I-42%, S- 63%, aa: H-I: 130  *Not found in Honeybee* |
|  | Muted | MUTED |  | C (*) | CNO, PLDN, BLOC1 |  |  | I-32%, S- 51%, aa: H-190, A-150  *Not found in Beetle and Mosquito* |
|  | Rab-Myosin 7A interacting | MYRIP | Rab effector MyRIP | C (*) | MYOVA, RAB27A | Cyto |  | aa: H-860  *Not in Insects* |
|  | Tetraspanin, Similar to CD151 | NET2 /  TSPAN12 | Tetraspanin-12 | C | DNM1, |  |  | I-41%, S-59%, aa:H-I:250  *Many isoforms in Beetle* |
|  | Neuroligin | NLGN2 | Neuroligin-2 precursor | C | PSD95 |  |  | I -33%, S- 50%, aa: H-840, T-950  3-4 paralogs in insects |
|  | Neurexin | NRXN1 |  | C | SYT1, LIN2, PSD95 |  |  | I-36%, S-55%, aa: H-1480, D-1840 |
|  | NEM-sensitive fusion protein | NSF |  | A | GOSR2, SNAPA, SNAREs | EZ | Exo | I-65%, S-80%, aa: H-I:740 |
|  | Protein kinase C and casein kinase substrate | PACSIN1 | Protein kinase C and casein kinase substrate in neurons protein 1 | A (*) | SYN1, SYNJ1, DNM1 |  | Endo | I-42%, S-61%, aa H-440, A-480  *Not in Beetle* |
|  | Piccolo | PCLO | Aczonin | C (*) | BSN, RIMS |  |  | I-27%, S-40%, aa: H-I: 5100  *No insects homologues* |
|  | Phosphatidylinositol-binding clathrin assembly protein | PICALM |  | C | AP2A, DNM1 |  | Endo | I-45%, S-58%, aa: H-I: 640 |
|  | Phosphatidylinositol  4 kinase | PIK4CA | Isoform 1 phosphatidylinositol 4- kinase alpha | B | SYNJN1 | EZ | Endo | I-49%, S-66%, aa: H-I 2060 |
|  | Phosphatidylinositol-4-phosphate 5-kinase | PIP5K1C | Phosphatidylinositol-4-phosphate 5-kinase type-1 γ | B | ARF6, Actin | EZ | Endo | I-55%, S-67%, aa: H-670, T-780 |
|  | Pallidin | PLDN |  | C (*) | CNO, BLOC1, MUTED |  |  | I-27%, S-48%, aa : H-170, A-140  *Not in Beetle. Fragment in Honeybee* |
|  | Liprin | PPFIA3 | Isoform 1 of Liprin-alpha-3 | A (*) | ERC2, RIMS1, GIT1 |  |  | I-63%, S-80%, aa:H-1190, T-340  *Not in honeybee* |
|  | Cytohesin-1 | PSCD1 | Pleckstrin homology, Sec7 and coiled-coil domains 1 | A | ARF-GEF, MUNC13 |  | Exo-Endo | I-70%, S-85%, aa: H-400, A-670 |
|  | Arno 2 | PSCD2 | Cytohesin-2  (ARNO ‎protein) (ARF ‎exchange factor) Cytohesin-2 (ARF ‎nucleotide-binding site ‎opener) (ARNO ‎protein) (ARF ‎exchange factor) (399 ‎aa)‎ | A | ARF1, ARF6 |  | Exo-Endo | I-67%, S-83%, aa:400, T-450 |
|  | Rab | RAB27A | Ras-related protein 27 | A | UNC13A, RPH3AL, |  | Exo | I-69%, S-84 %, aa : H-I:220 |
|  | Rab | RAB3A | Ras-related protein 3A | A | RPH3AL, RIMS1, UNC13 |  | Exo | I-80%, S-90%, aa: H-I: 220 |
|  | Rab3-Gap150 | RAB3GAP | RAB3 GTPase activating protein subunit | C (*) | UNC13, SYX1A, RIMS, EXOSC6 | EZ |  | I-30%, S-49%, aa: H-1390, T-1270  *Not detected in Honeybee* |
|  | Rabin 3 | RAB3IL1 | RAB3A interacting protein-like 1 | C (*) | RAB3A |  |  | I-43%, S-62%, aa: H-360, T-420  *Not in Mosquito and fly* |
|  | RAB6 interacting protein | RAB6IP1 | Rab6-interacting protein 1 | C | RAB6A |  |  | I-41%, S-57%, aa: H-1360, A-1320 |
|  | YIP3 homolog | RABAC1 | Prenylated Rab acceptor protein 1 | B | RAB, VAMP |  |  | I-44%, S-66%, aa: H-I:190 |
|  | Rab GTPase | RABGAP1 | Rab GTPase-activating protein 1 | B (*) | RAB6, KIF20 | EZ |  | I-48%, S-69%, aa: H-1000, A-820  *Not detected in Honeybee* |
|  | Ral | RALA | Ras-related protein Ral-A precursor | A | EXOC8, SEC5, CALM1 |  |  | I-70%, S-80%, aa: H-I:200 |
|  | Rap guanine nucleotide exchange factor | RAPGEF4 | Isoform 1 of Rap guanine nucleotide exchange factor 4 | B (*) | RIMS2, RAP1B, ATXN1 |  |  | I-51% , S-68%, aa: H-1010, T-930‎  *Not in Honeybee* |
|  | Rab-interacting protein | RILP | Isoform 2 of Rab-interacting lysosomal protein | C (*) | RABAC1, VAMP |  |  | *Not in insects.* H-190 |
|  | RimS binding protein | RIMBP2 | Isoform 2 of RIM-binding protein 2 | C | RIMS, CACNA |  |  | I-35%, S-54%, aa: H-650, A-1380 |
|  | Rims | RIMS1 | Regulating synaptic membrane exocytosis 1 | C | UNC13, CACNA, RAB3A, SYT1 |  | Exo-Endo | I-37%, S-50%, aa: H-1690, T-1480 |
|  | Rabphilin 3A | RPH3A | Isoform 1 of Rabphilin-3A | C | RAB3A, SYX1, NRXN1 |  | Exo | I-41%, S-63%, aa: H-690, T-740 |
|  | Stoned | SALF / STON /STNB | Stonin, Stoned B | C | EPS15, SYT1 |  | Endo | I-29%, S-47%, aa: H-740, T-1030 |
|  | SCAMP37 | SCAMP1 | Secretory carrier membrane protein 1 | C | SNAP23, VAMP3 |  |  | I-47%, S-62%, aa: H-I: 360 |
|  | Scinderin | SCIN | Isoform 1 of Adseverin | C (*) | Actin | Cyto |  | I-42%, S-59%, aa: H-I; 730  *Not in Honeybee and Beetle* |
|  | Sec22-like | SEC22B | Sec22 vesicle trafficking protein homolog B | A | BET1, STX5A |  | Exo | I-62%, S-%78, aa: H-I:210 |
|  | Septin 5 | SEPT5 | Peanut-like 1 | A | STX1A, STX4 |  | Exo | I-62%, S-79%, aa: H-I:370 |
|  | SH3-domain GRB2-like | SH3GL1 | Endophilin | B | BIN2, CLTC, SYNJ1 |  | Endo | I-53%, S-69%, aa:H-I: 370 |
|  | Signal-induced proliferation-associated protein | SIPA1L1 | Isoform 1 of Signal-induced proliferation-associated 1-like protein 1 | C | PSD95, NRXN1, NLGN2 |  |  | I -37%, S- 52%, aa: H-1800, T-1370  2 paralogs in insects |
|  | vGluT1 | SLC17A7 | Solute carrier family 17, member 7 | C | SYP, VGAT, ZN-T3 | M |  | I-42%, S- 62%, aa: H-560, T-500  *Insects: 12-14 genes and variants* |
|  | SNAP-25 | SNAP25 | Synaptosomal-associated protein 25 | A | SNAREs, SYT1  CACNA |  | Exo | I-61%, S-77%, aa: H-I::210 |
|  | SNAP-29 | SNAP29 | Synaptosomal-associated protein 29 | C | STX6, SYBL1 | M | Exo | I-34% , S-54%, aa: H-I: 230 |
|  | AP180 | SNAP91 | Synaptosomal-associated protein, 91kDa (LAP-insect) | C | SYP, AMPH, AP2 |  | Endo | I-37%, S-49%, aa: H- 910, A-860 |
|  | SNAP | SNAPA | α, β, γ-soluble NSF attachment protein | A | SNAREs, SYT1 |  | Exo | I-66%, S-84%, aa: H-I:290 |
|  | Snapin | SNAPAP | SNAP binding protein | B (*) | BLOC1, SNAP25 |  | Exo | I-48%, S-72%, aa: H:140, A-160  *Not in Beetle* |
|  | Snip | SNIP | SNAP-25-interacting protein,  P140 | C (*) | SNAP25 |  | Exo | I-32%, S-52%, aa: H-1050, D-790  *No insect homologues* |
|  | Syntaphilin | SNPH |  | C (*) | SNAP25, DNM1, SYX1A, SYP |  |  | aa: H: 540  *No insect homologues –weak to dynactin* |
|  | Sorting nexin | SNX9 | Sorting nexin-9 | C (*) | SYNJ1, CLTC |  | Endo | I-35% , S-54%, aa:H-600, A-540  *Not in Beetle* |
|  | Syntaxin | STX1A | HPC-1 | A | SNAREs, SYT1,  STXBP1, CACAN | M | Exo | I-73%, S-86%, aa: H-I: 290 |
|  | n-Sec | STXBP1 | Munc-18 | A | SYX1A, CDK5, SNAP25 |  | Exo | I-66%, S-79%, aa: H-I: 590 |
|  | Tomosyn | STXBP5 | Syntaxin binding protein 5 | B | SYX1A, SNAP23 |  | Exo | I-48%, S-65%, aa: H-I:1150  *Many isoforms in fly* |
|  | Amisyn | STXBP6 | Syntaxin binding protein 6 | C (*) | SNAREs |  |  | I-24%, S-47%, aa: H-210; A-860*No homologues, Weak to Exocyst Sec3* |
|  | SV2 | SV2A | Synaptic vesicle glycoprotein 1 | C | SYT1 | M | Exo | I-31%, S-51%, aa: H-740; A-590  *Multiple genes and isoforms (4-6)* |
|  | Synaptobrevin-like protein | SYBL1 | Isoform 1 of Synaptobrevin-like protein 1 | A | SYX7, SYX8, VAMP3, VAMP2 |  | Exo | I-59%, S-78%, aa: H-i: 220  *Insects - 3 genes* |
|  | Synapsin | SYN1 |  | C | SRC, CAMK1 | Cyto |  | I-43%, S-61%, aa: H-700; T-560 |
|  | Synaptogyrin | SYNGR1 | Cellugyrin like | B (*) | SYP | M |  | I-51%, S-66%, aa: H-I: 230  *Not in Honeybee* |
|  | Synaptojanin | SYNJ1 |  | B | ESP15, AMPH PIK4CA, | EZ | Endo | I-53%, S-68%, aa: H-1570, A-1150 |
|  | Synaptoporin | SYNPR |  | C (*) | SNAP25, VAMP3, SCAMP | M |  | aa: H- 280  *Not in Insects. Only remote in Beetle* |
|  | Synaptophysin | SYP | Major synaptic vesicle protein p38 | C (*) | VAMP2, SRC | M | Exo | aa: H- 310  *Not in Insects* |
|  | Synaptotagmin | SYT1 | P65 | A | SNARE, CACNA, SV2, AP2 | M | Exo | I-64%, S-78%, aa: H-430, A-400 |
|  | Synaptotagmin | SYT5 | Synaptotagmin V | A | NRXN1, SNAP25 | M | Exo | I-58%, S-77%, aa: H-390, A-430 |
|  | Synaptotagmin | SYT9 | Synaptotagmin IX | B (*) | SNAP25, STX1A | M | Exo | I-53%, S-69%, aa: H-I:490  *Not in Honeybee, Mosquito or Fly* |
|  | Granulophilin | SYTL4 | Exophilin-2 | C (*) | SYT1A, STXBP1, RAB3A |  | Exo-Endo | I-43%, S-61%, aa: H-670, A-710  *Not homologous, C2 domains conserved* |
|  | Synaptotagmin-like protein 5‎ | SYTL5 |  | C (*) | RAB3, RAB6, RAB27, UNC13D |  | Exo | I-38%, S-52%, aa: H-730, A-2120  *Not homologous, C2 domains conserved* |
|  | SV31 | TMEM163 | Isoform 1 of Transmembrane protein 163 | C (*) |  | M |  | aa: H -290  *Not in Insects* |
|  | Bet5 homolog | TRAPPC1 | Trafficking protein particle complex 1, Sybindin-like | A | BET1 |  | Exo | I-54%, S-79%, aa: H-I:150 |
|  | Sybindin | TRAPPC4 | Trafficking protein particle complex subunit | A | SDC2 |  |  | I-61%, S-80%, aa: H-I: 220 |
|  | Alpha-taxilin | TXLNA |  | C (*) | SYNX1A, SYN4A |  | Exo | I-40%, S-65%, aa: H-550, A-360  *Not in Honeybee* |
|  | Munc-13 | UNC13B |  | A (*) | RIM, DOC2, SYX1, SNAP25 |  | Exo-Endo | I-68%, S-81%, aa: H-1590, D-2870  *Not in Honeybee or Anopheles* |
|  | Unc-13 homolog | UNC13D | Unc-13 homolog D | C | RAB27A, SYX11, SYTL5 |  | Exo-Endo | I-29%, S-46%, aa H-1090, T-1260 |
|  | VAMP | VAMP2 | Synaptobrevin 2 | A | SNAREs, SYP, UNC13B, SYT1 | M | Exo | I-72%, S-80%, aa: H-I:120 |
|  | VAP33 | VAPA | VAMP-associated protein A, 33Kda | B | VAMPs, SNAP23 |  | Exo | I-43%, S-65%, aa: H-I: 250  *Many isoforms in fly* |
|  | VAT-1 | VAT1 | Vesicle transport protein 1 homolog | C (*) |  |  |  | I-46%, S-63%, aa: H-390, A-370  *Not in Fly. Strongly conserved* |
|  | Vacuolar protein sorting 18 homolog | VPS18 | Vacuolar protein sorting 18 homolog | C | SYX6, SYX7, NSF |  |  | I-34%, S-54%, aa: H-I:970 |
|  | Vps-33B | VPS33B | Vacuolar protein sorting 33B | C | NSF |  |  | I-31%, S-51%,aa: H-620, T-590  *In fly and Honeybee- 3 genes* |
|  | Vti1 | VTI1B | Vesicle transport v-SNARE protein Vti1-like 1 | C | VAMP8, SYX12 |  | Exo | I-30%, S-55%, aa: H-I: 230 |
|  | 14-3-3 | YWHAQ | 14-3-3 protein theta | A | RAF1 | ST |  | I-76%, S-86%, aa: H-I: 250 |

**Additional data file 2B**

| **Protein AC** | **Protein ID** | **Protein Name** | **Length** | **Keyword** | **Pfam Name** |
| --- | --- | --- | --- | --- | --- |
| P27348 | 1433T_HUMAN | 14-3-3 protein theta (14-3-3 protein tau) (14-3-3 protein T-cell) (HS1 protein) | 245 | 3d-structure; cytoplasm; direct protein sequencing | 14-3-3 protein |
| A6H8W6 | A6H8W6_HUMAN | SIPA1L1 protein | 1803 |  |  |
| P35612 | ADDB_HUMAN | Beta-adducin (Erythrocyte adducin subunit beta) | 726 | acetylation; alternative splicing; calmodulin-binding; cytoplasm; cytoskeleton; direct protein sequencing; membrane; phosphorylation; polymorphism | Class II Aldolase and Adducin N-terminal domain |
| Q9Y6U3 | ADSV_HUMAN | Adseverin (Scinderin) | 715 | actin capping; actin-binding; alternative splicing; calcium; cytoplasm; cytoskeleton; repeat | Gelsolin repeat |
| P49418 | AMPH_HUMAN | Amphiphysin | 695 | 3d-structure; alternative splicing; cell junction; **coiled coil**; cytoplasm; cytoplasmic vesicle; cytoskeleton; membrane; phosphorylation; sh3 domain; synapse | SH3 domain; BAR domain |
| O60641 | AP180_HUMAN | Clathrin coat assembly protein AP180 (Clathrin coat-associated protein AP180) (91 kDa synaptosomal-associated protein) | 907 | alternative splicing; coated pit; membrane; nitration; phosphorylation | ANTH domain |
| O95782 | AP2A1_HUMAN | AP-2 complex subunit alpha-1 (Adapter-related protein complex 2 alpha- 1 subunit) (Alpha-adaptin A) (Adaptor protein complex AP-2 alpha-1 subunit) (Clathrin assembly protein complex 2 alpha-A large chain) (100 kDa coated vesicle protein A) (Plasma membran | 977 | alternative splicing; coated pit; cytoplasmic vesicle; endocytosis; golgi apparatus; membrane; phosphorylation; protein transport; transport | Adaptin N terminal region; Alpha adaptin AP2, C-terminal domain; Adaptin C-terminal domain |
| O14617 | AP3D1_HUMAN | AP-3 complex subunit delta-1 (Adapter-related protein complex 3 subunit delta-1) (Delta-adaptin 3) (AP-3 complex subunit delta) (Delta-adaptin) | 1153 | alternative splicing; golgi apparatus; phosphorylation; polymorphism; protein transport; transport | Adaptin N terminal region; Bovine leukaemia virus receptor |
| Q02410 | APBA1_HUMAN | Amyloid beta A4 precursor protein-binding family A member 1 (Neuron- specific X11 protein) (Neuronal Munc18-1-interacting protein 1) (Mint- 1) (Adapter protein X11alpha) | 837 | 3d-structure; protein transport; repeat; transport | PDZ domain; Phosphotyrosine interaction domain |
| Q99767 | APBA2_HUMAN | Amyloid beta A4 precursor protein-binding family A member 2 (Neuron- specific X11L protein) (Neuronal Munc18-1-interacting protein 2) (Mint-2) (Adapter protein X11beta) | 749 | phosphorylation; protein transport; repeat; transport | PDZ domain; Phosphotyrosine interaction domain |
| P84077 | ARF1_HUMAN | ADP-ribosylation factor 1 | 181 | 3d-structure; P-loop; blocked amino end; er-golgi transport; golgi apparatus; gtp-binding; lipoprotein; myristate; myristylation; nucleotide binding; nucleotide-binding; protein transport; transport | ADP-ribosylation factor family |
| P62330 | ARF6_HUMAN | ADP-ribosylation factor 6 | 175 | 3d-structure; P-loop; blocked amino end; er-golgi transport; golgi apparatus; gtp-binding; lipoprotein; myristate; myristylation; nucleotide binding; nucleotide-binding; protein transport; transport | ADP-ribosylation factor family |
| P53365 | ARFP2_HUMAN | Arfaptin-2 (ADP-ribosylation factor-interacting protein 2) (Partner of RAC1) (Protein POR1) | 341 | 3d-structure; leucine zipper | Arfaptin-like domain |
| O94812 | BAIP3_HUMAN | BAI1-associated protein 3 (BAP3) | 1187 | alternative splicing; polymorphism; repeat | C2 domain; Domain of Unknown Function |
| O15155 | BET1_HUMAN | BET1 homolog (Golgi vesicular membrane-trafficking protein p18) (hBET1) | 118 | **coiled** coil; endoplasmic reticulum; er-golgi transport; golgi apparatus; membrane; phosphorylation; protein transport; transmembrane; transport | SNARE domain |
| Q9Y6D5 | BIG2_HUMAN | Brefeldin A-inhibited guanine nucleotide-exchange protein 2 (Brefeldin A-inhibited GEP 2) | 1785 | guanine-nucleotide releasing factor; phosphorylation; polymorphism | Sec7 domain; Domain of unknown function |
| O00499 | BIN1_HUMAN | Myc box-dependent-interacting protein 1 (Bridging integrator 1) (Amphiphysin-like protein) (Amphiphysin II) (Box-dependent myc- interacting protein 1) | 593 | 3d-structure; alternative splicing; anti-oncogene; cell cycle; **coiled** coil; cytoplasm; developmental protein; differentiation; endocytosis; host-virus interaction; nucleus; phosphorylation; sh3 domain | SH3 domain; BAR domain |
| P78537 | BL1S1_HUMAN | Biogenesis of lysosome-related organelles complex-1 subunit 1 (BLOC-1 subunit 1) (GCN5-like protein 1) (RT14 protein) | 125 | brain | GCN5-like protein 1 |
| Q9UPA5 | BSN_HUMAN | Protein bassoon (Zinc finger protein 231) | 3926 | cell junction; **coiled** coil; cytoplasm; cytoskeleton; glycoprotein; lipoprotein; metal-binding; myristate; phosphorylation; repeat; synapse; synaptosome; zinc; zinc-finger | Piccolo Zn-finger |
| O00555 | CAC1A_HUMAN | Voltage-dependent P/Q-type calcium channel subunit alpha-1A (Voltage- gated calcium channel subunit alpha Cav2.1) (Calcium channel, L type, alpha-1 polypeptide isoform 4) (Brain calcium channel I) (BI) | 2505 | alternative splicing; calcium; calcium channel; calcium transport; disease mutation; glycoprotein; ion transport; ionic channel; membrane; neurodegeneration; phosphorylation; polymorphism; repeat; spinocerebellar ataxia; transmembrane; transport; triplet | Ion transport protein; Voltage gated calcium channel IQ domain |
| P62158 | CALM_HUMAN | Calmodulin (CaM) | 149 | 3d-structure; EF hand; acetylated amino end; acetylation; calcium; calcium binding; direct protein sequencing; duplication; methylated amino acid; methylation; phosphorylation; repeat; ubl conjugation | EF hand |
| Q9ULU8 | CAPS1_HUMAN | Calcium-dependent secretion activator 1 (Calcium-dependent activator protein for secretion 1) (CAPS-1) | 1353 | 3d-structure; alternative splicing; calcium; cell junction; cytoplasmic vesicle; exocytosis; lipid-binding; membrane; metal-binding; protein transport; synapse; transport | C2 domain; PH domain; Domain of Unknown Function |
| Q00610 | CLH1_HUMAN | Clathrin heavy chain 1 (CLH-17) | 1675 | alternative splicing; coated pit; cytoplasmic vesicle; direct protein sequencing; membrane; phosphorylation | Region in Clathrin and VPS; Clathrin propeller repeat; Clathrin, heavy-chain linker |
| Q9NUP1 | CNO_HUMAN | Protein cappuccino homolog | 217 | **coiled** coil; cytoplasm |  |
| P78357 | CNTP1_HUMAN | Contactin-associated protein 1 precursor (Caspr) (Caspr1) (Neurexin 4) (Neurexin IV) (p190) | 1384 | cell adhesion; egf-like domain; glycoprotein; membrane; phosphorylation; repeat; sh3-binding; signal; transmembrane | EGF-like domain; Fibrinogen beta and gamma chains, C-terminal globular domain; F5/8 type C domain; Laminin G domain |
| Q6PUV4 | CPLX2_HUMAN | Complexin-2 (Complexin II) (CPX II) (Synaphin-1) | 134 | **coiled** coil; cytoplasm; exocytosis; mast cell degranulation; membrane trafficking; neurotransmitter transport; phosphorylation; transport | Synaphin protein |
| O14936 | CSKP_HUMAN | Peripheral plasma membrane protein CASK (EC 2.7.11.1) (hCASK) (Calcium/calmodulin-dependent serine protein kinase) (Lin-2 homolog) | 926 | 3d-structure; alternative splicing; atp-binding; calmodulin-binding; cytoplasm; kinase; magnesium; membrane; metal-binding; nucleotide-binding; nucleus; repeat; serine/threonine-protein kinase; sh3 domain; transferase | Protein kinase domain; PDZ domain; Guanylate kinase; L27 domain; Variant SH3 domain |
| Q15438 | CYH1_HUMAN | Cytohesin-1 (PH, SEC7 and coiled-coil domain-containing protein 1) (SEC7 homolog B2-1) | 398 | 3d-structure; alternative splicing; **coiled** coil; guanine-nucleotide releasing factor | PH domain; Sec7 domain |
| Q99418 | CYH2_HUMAN | Cytohesin-2 (PH, SEC7 and coiled-coil domain-containing protein 2) (ARF nucleotide-binding site opener) (Protein ARNO) (ARF exchange factor) | 400 | 3d-structure; alternative splicing; **coiled** coil; guanine-nucleotide releasing factor | PH domain; Sec7 domain |
| Q12959 | DLG1_HUMAN | Disks large homolog 1 (Synapse-associated protein 97) (SAP-97) (hDlg) | 904 | 3d-structure; alternative splicing; cell junction; duplication; endoplasmic reticulum; host-virus interaction; membrane; phosphorylation; postsynaptic cell membrane; repeat; sh3 domain; synapse | SH3 domain; PDZ domain; Guanylate kinase; L27_1 |
| Q9H3Z4 | DNJC5_HUMAN | DnaJ homolog subfamily C member 5 (Cysteine string protein) (CSP) | 198 | alternative splicing; chaperone; lipoprotein; membrane; palmitate; phosphorylation; thiolester bond | DnaJ domain |
| Q14184 | DOC2B_HUMAN | Double C2-like domain-containing protein beta (Doc2-beta) | 412 | calcium; calcium/phospholipid-binding; membrane; repeat | C2 domain |
| Q05193 | DYN1_HUMAN | Dynamin-1 (EC 3.6.5.5) | 864 | 3d-structure; GTP binding; P-loop; alternative splicing; cytoplasm; cytoskeleton; endocytosis; gtp-binding; hydrolase; membrane trafficking; microtubule; motor protein; nucleotide binding; nucleotide-binding; phosphorylation | PH domain; Dynamin family; Dynamin central region; Dynamin GTPase effector domain |
| Q9H4M9 | EHD1_HUMAN | EH domain-containing protein 1 (Testilin) (hPAST1) | 534 | atp-binding; calcium; **coiled** coil; direct protein sequencing; endosome; membrane; nucleotide-binding; phosphorylation | Dynamin family |
| P42566 | EP15_HUMAN | Epidermal growth factor receptor substrate 15 (Protein Eps15) (AF-1p protein) | 896 | 3d-structure; calcium; chromosomal rearrangement; cytoplasm; membrane; phosphorylation; polymorphism; proto-oncogene; repeat; sh3-binding | EF hand |
| Q9Y6I3 | EPN1_HUMAN | Epsin-1 (EPS-15-interacting protein 1) (EH domain-binding mitotic phosphoprotein) | 551 | 3d-structure; alternative splicing; coated pit; cytoplasm; endocytosis; lipid-binding; membrane; nucleus; phosphorylation; repeat; ubl conjugation | ENTH domain; Ubiquitin interaction motif |
| Q8TAG9 | EXOC6_HUMAN | Exocyst complex component 6 (Exocyst complex component Sec15A) (Sec15- like 1) | 804 | exocytosis; protein transport; transport | Exocyst complex subunit Sec15-like |
| P50395 | GDIB_HUMAN | Rab GDP dissociation inhibitor beta (Rab GDI beta) (Guanosine diphosphate dissociation inhibitor 2) (GDI-2) | 445 | cytoplasm; gtpase activation; membrane | GDP dissociation inhibitor |
| Q9HD26 | GOPC_HUMAN | Golgi-associated PDZ and coiled-coil motif-containing protein (PDZ protein interacting specifically with TC10) (PIST) (CFTR-associated ligand) (Fused in glioblastoma) | 462 | alternative splicing; cell junction; cell projection; chromosomal rearrangement; **coiled** coil; cytoplasm; golgi apparatus; membrane; phosphorylation; postsynaptic cell membrane; protein transport; synapse; transport | PDZ domain |
| O14653 | GOSR2_HUMAN | Golgi SNAP receptor complex member 2 (27 kDa Golgi SNARE protein) (Membrin) | 212 | alternative splicing; **coiled** coil; golgi apparatus; membrane; polymorphism; protein transport; transmembrane; transport | Vesicle transport v-SNARE protein |
| O14964 | HGS_HUMAN | Hepatocyte growth factor-regulated tyrosine kinase substrate (Protein pp110) (Hrs) | 777 | 3d-structure; cytoplasm; endosome; membrane; metal-binding; phosphorylation; protein transport; transport; zinc; zinc-finger | VHS domain; FYVE zinc finger; Ubiquitin interaction motif |
| Q9NZM3 | ITSN2_HUMAN | Intersectin-2 (SH3 domain-containing protein 1B) (SH3P18) (SH3P18-like WASP-associated protein) | 1696 | 3d-structure; alternative splicing; calcium; **coiled** coil; cytoplasm; endocytosis; phosphorylation; polymorphism; repeat; sh3 domain | SH3 domain; EF hand; C2 domain; PH domain; RhoGEF domain; Variant SH3 domain |
| Q12756 | KIF1A_HUMAN | Kinesin-like protein KIF1A (Axonal transporter of synaptic vesicles) (Microtubule-based motor KIF1A) (UNC-104-and KIF1A-related protein) (hUNC-104) | 1690 | alternative splicing; atp-binding; **coiled** coil; microtubule; motor protein; nucleotide-binding | PH domain; Kinesin motor domain; FHA domain |
| P11279 | LAMP1_HUMAN | Lysosome-associated membrane glycoprotein 1 precursor (LAMP-1) (CD107a antigen) | 416 | direct protein sequencing; endosome; glycoprotein; lysosome; membrane; signal; transmembrane; transmembrane protein | Lysosome-associated membrane glycoprotein |
| O14910 | LIN7A_HUMAN | Lin-7 homolog A (Lin-7A) (hLin-7) (Mammalian lin-seven protein 1) (MALS-1) (Vertebrate lin-7 homolog 1) (Veli-1 protein) (Tax interaction protein 33) (TIP-33) | 233 | cell junction; exocytosis; membrane; postsynaptic cell membrane; protein transport; synapse; synaptosome; tight junction; transport | PDZ domain; L27 domain |
| O75145 | LIPA3_HUMAN | Liprin-alpha-3 (Protein tyrosine phosphatase receptor type f polypeptide-interacting protein alpha-3) (PTPRF-interacting protein alpha-3) | 1194 | alternative splicing; **coiled** coil; cytoplasm; phosphorylation; polymorphism; repeat | SAM domain; SAM domain |
| O94910 | LPHN1_HUMAN | Latrophilin-1 precursor (Calcium-independent alpha-latrotoxin receptor 1) (Lectomedin-2) | 1474 | alternative splicing; g-protein coupled receptor; glycoprotein; lectin; membrane; phosphorylation; receptor; signal; transducer; transmembrane | 7 transmembrane receptor; Latrophilin/CL-1-like GPS domain; Galactose binding lectin domain; Olfactomedin-like domain; Latrophilin Cytoplasmic C-terminal region; Hormone receptor domain |
| P16109 | LYAM3_HUMAN | P-selectin precursor (Granule membrane protein 140) (GMP-140) (PADGEM) (Leukocyte-endothelial cell adhesion molecule 3) (LECAM3) (CD62P antigen) | 830 | 3d-structure; cell adhesion; egf-like domain; glycoprotein; lectin; lipoprotein; membrane; palmitate; phosphohistidine; phosphoprotein; polymorphism; repeat; signal; surface antigen; sushi; transmembrane; transmembrane protein | EGF-like domain; Lectin C-type domain; Sushi domain |
| P47224 | MSS4_HUMAN | Guanine nucleotide exchange factor MSS4 (Rab-interacting factor) | 123 | 3d-structure; guanine-nucleotide releasing factor; metal-binding; protein transport; transport; zinc | Mss4 protein |
| Q8TDH9 | MUTED_HUMAN | Muted protein homolog | 187 | alternative splicing; **coiled** coil |  |
| Q8NFW9 | MYRIP_HUMAN | Rab effector MyRIP (Myosin-VIIa- and Rab-interacting protein) (Exophilin-8) (Slp homolog lacking C2 domains c) (SlaC2-c) | 859 | actin-binding; cytoplasm; metal-binding; phosphorylation; repeat; zinc; zinc-finger | Myelin-associated oligodendrocytic basic protein |
| P17677 | NEUM_HUMAN | Neuromodulin (Axonal membrane protein GAP-43) (Growth-associated protein 43) (PP46) (Neural phosphoprotein B-50) | 238 | calmodulin-binding; cell junction; cell projection; developmental protein; differentiation; growth regulation; lipoprotein; membrane; neurogenesis; palmitate; phosphoprotein; phosphorylation; polymorphism; synapse; thiolester bond | IQ calmodulin-binding motif; Neuromodulin |
| Q8NFZ4 | NLGN2_HUMAN | Neuroligin-2 precursor | 835 | cell adhesion; glycoprotein; membrane; phosphorylation; signal; transmembrane | Carboxylesterase |
| Q9ULB1 | NRX1A_HUMAN | Neurexin-1-alpha precursor (Neurexin I-alpha) | 1477 | alternative promoter usage; alternative splicing; calcium; cell adhesion; egf-like domain; glycoprotein; membrane; metal-binding; repeat; signal; transmembrane | EGF-like domain; Laminin G domain |
| P46459 | NSF_HUMAN | Vesicle-fusing ATPase (EC 3.6.4.6) (Vesicular-fusion protein NSF) (N- ethylmaleimide sensitive fusion protein) (NEM-sensitive fusion protein) | 744 | P-loop; atp-binding; cytoplasm; endoplasmic reticulum; golgi apparatus; hydrolase; magnesium; metal-binding; nucleotide binding; nucleotide-binding; polymorphism; protein transport; repeat; transport | ATPase family associated with various cellular activities; Cell division protein 48; Cell division protein 48 |
| Q9BY11 | PACN1_HUMAN | Protein kinase C and casein kinase substrate in neurons protein 1 | 444 | **coiled** coil; cytoplasm; endocytosis; phosphorylation; sh3 domain | SH3 domain; Fes/CIP4 homology domain |
| Q9Y6V0 | PCLO_HUMAN | Protein piccolo (Aczonin) | 5183 | 3d-structure; alternative splicing; calcium; calcium/phospholipid-binding; cell junction; metal-binding; phosphorylation; repeat; synapse; zinc; zinc-finger | C2 domain; Piccolo Zn-finger |
| P42356 | PI4KA_HUMAN | Phosphatidylinositol 4-kinase alpha (EC 2.7.1.67) (PI4-kinase alpha) (PtdIns-4-kinase alpha) (PI4K-alpha) | 2044 | alternative splicing; kinase; phosphotransferase; transferase | Phosphatidylinositol 3- and 4-kinase; Phosphoinositide 3-kinase family, accessory domain |
| O60331 | PI51C_HUMAN | Phosphatidylinositol-4-phosphate 5-kinase type-1 gamma (EC 2.7.1.68) (Phosphatidylinositol-4-phosphate 5-kinase type I gamma) (PtdIns(4)P- 5-kinase gamma) (PtdInsPKIgamma) (PIP5KIgamma) | 668 | kinase; membrane; phosphorylation; transferase | Phosphatidylinositol-4-phosphate 5-Kinase |
| Q13492 | PICAL_HUMAN | Phosphatidylinositol-binding clathrin assembly protein (Clathrin assembly lymphoid myeloid leukemia protein) | 652 | alternative splicing; chromosomal rearrangement; coated pit; cytoplasmic vesicle; endocytosis; golgi apparatus; membrane; polymorphism; proto-oncogene | ANTH domain |
| Q9UL45 | PLDN_HUMAN | Pallidin (Pallid protein homolog) (Syntaxin 13-interacting protein) | 172 | alternative splicing; **coiled** coil; cytoplasm; membrane; phosphorylation |  |
| Q9UI14 | PRAF1_HUMAN | Prenylated Rab acceptor protein 1 (PRA1 family protein 1) | 185 | cell junction; cytoplasm; cytoplasmic vesicle; golgi apparatus; membrane; synapse; transmembrane | PRA1 family protein |
| Q96MT3 | PRIC1_HUMAN | Prickle-like protein 1 (REST/NRSF-interacting LIM domain protein 1) | 831 | cytoplasm; lim domain; lipoprotein; membrane; metal-binding; nucleus; prenylation; repeat; zinc | LIM domain; PET Domain |
| Q149M6 | Q149M6_HUMAN | Exophilin 5 | 1989 |  |  |
| Q6FGM0 | Q6FGM0_HUMAN | SH3GL1 protein (SH3 domain GRB2-like 1) | 368 | sh3 domain | SH3 domain; BAR domain |
| Q86V32 | Q86V32_HUMAN | RAB3IL1 protein | 356 |  | GDP/GTP exchange factor Sec2p |
| Q9P2U7 | Q9P2U7_HUMAN | Brain-specific Na-dependent inorganic phosphate cotransporter | 560 |  | Major Facilitator Superfamily |
| Q6IQ26 | RA6I1_HUMAN | Rab6-interacting protein 1 (Rab6IP1) | 1287 | polymorphism; repeat | PLAT/LH2 domain; DENN; RUN domain; dDENN domain; uDENN domain |
| P20336 | RAB3A_HUMAN | Ras-related protein Rab-3A | 220 | GTP binding; P-loop; exocytosis; gtp-binding; lipoprotein; membrane; membrane trafficking; methylation; nucleotide binding; nucleotide-binding; phosphorylation; prenylated cysteine; prenylation; protein transport; transport | Ras family |
| P11233 | RALA_HUMAN | Ras-related protein Ral-A precursor | 206 | 3d-structure; GTP binding; P-loop; gtp-binding; host-virus interaction; lipoprotein; membrane; membrane protein; methylated carboxyl end; methylation; nucleotide binding; nucleotide-binding; prenylated cysteine; prenylation; proto-oncogene; transforming p | Ras family |
| P51159 | RB27A_HUMAN | Ras-related protein Rab-27A (Rab-27) (GTP-binding protein Ram) | 221 | GTP binding; P-loop; alternative splicing; direct protein sequencing; disease mutation; gtp-binding; lipoprotein; membrane; methylation; nucleotide binding; nucleotide-binding; polymorphism; prenylated cysteine; prenylation | Ras family |
| Q15042 | RB3GP_HUMAN | Rab3 GTPase-activating protein catalytic subunit (RAB3 GTPase- activating protein 130 kDa subunit) (Rab3-GAP p130) (Rab3-GAP) | 981 | alternative splicing; cytoplasm; gtpase activation; phosphorylation |  |
| Q8IUD2 | RB6I2_HUMAN | ELKS/RAB6-interacting/CAST family member 1 (RAB6-interacting protein 2) (ERC protein 1) | 1116 | alternative splicing; chromosomal rearrangement; **coiled** coil; cytoplasm; golgi apparatus; membrane; phosphorylation; protein transport; transport | FIP domain |
| Q9H2M9 | RBGPR_HUMAN | Rab3 GTPase-activating protein non-catalytic subunit (Rab3 GTPase- activating protein 150 kDa subunit) (Rab3-GAP p150) (Rab3-GAP regulatory subunit) (RAB3-GAP150) (RGAP-iso) | 1393 | alternative splicing; cataract; cytoplasm; disease mutation; gtpase activation; phosphorylation; polymorphism |  |
| O15034 | RIMB2_HUMAN | RIM-binding protein 2 (RIM-BP2) | 1052 | 3d-structure; alternative splicing; cell junction; membrane; phosphorylation; repeat; sh3 domain; synapse; ubl conjugation | Fibronectin type III domain; Variant SH3 domain |
| Q86UR5 | RIMS1_HUMAN | Regulating synaptic membrane exocytosis protein 1 (Rab3-interacting molecule 1) (RIM 1) | 1692 | 3d-structure; alternative splicing; cell junction; cone-rod dystrophy; disease mutation; exocytosis; metal-binding; neurotransmitter transport; phosphorylation; repeat; sensory transduction; synapse; transport; vision; zinc; zinc-finger | C2 domain; PDZ domain |
| Q9Y2J0 | RP3A_HUMAN | Rabphilin-3A (Exophilin-1) | 694 | alternative splicing; cell junction; metal-binding; phosphorylation; protein transport; repeat; synapse; transport; zinc; zinc-finger | C2 domain; Rabphilin-3A effector domain |
| Q8WZA2 | RPGF4_HUMAN | Rap guanine nucleotide exchange factor 4 (cAMP-regulated guanine nucleotide exchange factor II) (cAMP-GEFII) (Exchange factor directly activated by cAMP 2) (Epac 2) | 1011 | alternative promoter usage; alternative splicing; camp; camp-binding; cytoplasm; exocytosis; guanine-nucleotide releasing factor; membrane; nucleotide-binding; repeat | Cyclic nucleotide-binding domain; Domain found in Dishevelled, Egl-10, and Pleckstrin; RasGEF domain; Guanine nucleotide exchange factor for Ras-like GTPases; N-terminal motif; Ras association |
| O95295 | S25BP_HUMAN | SNARE-associated protein Snapin (Synaptosomal-associated protein 25- binding protein) (SNAP-associated protein) | 136 | cell junction; **coiled** coil; cytoplasm; cytoplasmic vesicle; exocytosis; membrane; phosphorylation; polymorphism; synapse; synaptosome |  |
| O75396 | SC22B_HUMAN | Vesicle-trafficking protein SEC22b (SEC22 vesicle-trafficking protein homolog B) (SEC22 vesicle-trafficking protein-like 1) (ERS24) (ERS- 24) | 215 | **coiled** coil; direct protein sequencing; endoplasmic reticulum; er-golgi transport; golgi apparatus; membrane; phosphorylation; protein transport; transmembrane; transport | Synaptobrevin |
| O15126 | SCAM1_HUMAN | Secretory carrier-associated membrane protein 1 (Secretory carrier membrane protein 1) | 338 | alternative splicing; membrane; protein transport; transmembrane; transport | SCAMP family |
| Q99719 | SEPT5_HUMAN | Septin-5 (Peanut-like protein 1) (Cell division control-related protein 1) (CDCrel-1) | 369 | cell cycle; cell division; **coiled** coil; direct protein sequencing; gtp-binding; nucleotide-binding; phosphorylation | Septin |
| P54920 | SNAA_HUMAN | Alpha-soluble NSF attachment protein (SNAP-alpha) (N-ethylmaleimide- sensitive factor attachment protein, alpha) | 295 | er-golgi transport; membrane; protein transport; transport | Aromatic-di-Alanine |
| O43759 | SNG1_HUMAN | Synaptogyrin-1 | 233 | alternative splicing; cell junction; membrane; synapse; transmembrane | Membrane-associating domain |
| Q9C0H9 | SNIP_HUMAN | p130Cas-associated protein (p140Cap) (SNAP-25-interacting protein) (SNIP) | 1055 | alternative splicing; **coiled** coil; cytoplasm; cytoskeleton; direct protein sequencing; exocytosis; phosphorylation |  |
| P60880 | SNP25_HUMAN | Synaptosomal-associated protein 25 (SNAP-25) (Synaptosomal-associated 25 kDa protein) (Super protein) (SUP) | 206 | 3d-structure; alternative splicing; cell junction; **coiled** coil; lipoprotein; palmitate; phosphorylation; repeat; synapse; synaptosome | SNAP-25 family; SNARE domain |
| O95721 | SNP29_HUMAN | Synaptosomal-associated protein 29 (SNAP-29) (Vesicle-membrane fusion protein SNAP-29) (Soluble 29 kDa NSF attachment protein) | 258 | cell junction; **coiled** coil; cytoplasm; membrane; protein transport; synapse; synaptosome; transport | SNAP-25 family; SNARE domain |
| O15079 | SNPH_HUMAN | Syntaphilin | 538 | cell junction; **coiled** coil; membrane; phosphorylation; synapse; synaptosome; transmembrane |  |
| Q9Y5X1 | SNX9_HUMAN | Sorting nexin-9 (SH3 and PX domain-containing protein 1) (Protein SDP1) (SH3 and PX domain-containing protein 3A) | 595 | phosphorylation; protein transport; sh3 domain; transport | SH3 domain; PX domain |
| Q9Y6Q2 | STON1_HUMAN | Stonin-1 (Stoned B-like factor) | 735 | cytoplasm; endocytosis; membrane; polymorphism | Adaptor complexes medium subunit family |
| Q16623 | STX1A_HUMAN | Syntaxin-1A (Neuron-specific antigen HPC-1) | 288 | alternative splicing; **coiled** coil; membrane; neurotransmitter transport; phosphorylation; secreted; transmembrane; transport; williams-beuren syndrome | Syntaxin; SNARE domain |
| P61764 | STXB1_HUMAN | Syntaxin-binding protein 1 (Unc-18 homolog) (Unc-18A) (Unc-18-1) (N- Sec1) (p67) | 594 | alternative splicing; cytoplasm; membrane; phosphorylation; protein transport; transport | Sec1 family |
| Q5T5C0 | STXB5_HUMAN | Syntaxin-binding protein 5 (Tomosyn-1) (Lethal(2) giant larvae protein homolog 3) | 1151 | alternative splicing; cell junction; **coiled** coil; cytoplasm; cytoplasmic vesicle; membrane; phosphorylation; protein transport; repeat; synapse; transport; wd repeat | WD domain, G-beta repeat; LLGL2 |
| Q8NFX7 | STXB6_HUMAN | Syntaxin-binding protein 6 (Amisyn) | 210 | alternative splicing; **coiled** coil; cytoplasm; membrane |  |
| Q7L0J3 | SV2A_HUMAN | Synaptic vesicle glycoprotein 2A | 742 | alternative splicing; cell junction; cytoplasmic vesicle; glycoprotein; membrane; neurotransmitter transport; phosphorylation; synapse; transmembrane; transport | Major Facilitator Superfamily |
| P51809 | SYBL1_HUMAN | Synaptobrevin-like protein 1 (Tetanus insensitive VAMP) (Ti-VAMP) (Ti- VAMP/VAMP7) | 220 | acetylation; alternative splicing; **coiled** coil; direct protein sequencing; endoplasmic reticulum; golgi apparatus; membrane; protein transport; transmembrane; transport | Synaptobrevin |
| Q9NX95 | SYBU_HUMAN | Syntabulin (Syntaxin-1-binding protein) (Golgi-localized syntaphilin- related protein) | 663 | alternative splicing; **coiled** coil; golgi apparatus; membrane; phosphorylation; transmembrane |  |
| P17600 | SYN1_HUMAN | Synapsin-1 (Synapsin I) (Brain protein 4.1) | 705 | actin binding; actin-binding; alternative splicing; cell junction; epilepsy; glycoprotein; phosphoprotein; phosphorylation; repeat; synapse | Synapsin, N-terminal domain; Synapsin, ATP binding domain |
| O43426 | SYNJ1_HUMAN | Synaptojanin-1 (EC 3.1.3.36) (Synaptic inositol-1,4,5-trisphosphate 5- phosphatase 1) | 1575 | 3d-structure; alternative splicing; cytoplasm; endocytosis; hydrolase; phosphorylation; repeat; rna-binding | SacI homology domain; Endonuclease/Exonuclease/phosphatase family; Domain of unknown function |
| Q8TBG9 | SYNPR_HUMAN | Synaptoporin | 265 | cell junction; cytoplasmic vesicle; glycoprotein; membrane; repeat; synapse; synaptosome; transmembrane | Membrane-associating domain |
| P08247 | SYPH_HUMAN | Synaptophysin (Major synaptic vesicle protein p38) | 313 | calcium; cell junction; cytoplasmic vesicle; glycoprotein; membrane; membrane trafficking; repeat; synapse; synaptosome; tandem repeat; transmembrane; transmembrane protein; ubl conjugation | Membrane-associating domain |
| P21579 | SYT1_HUMAN | Synaptotagmin-1 (Synaptotagmin I) (SytI) (p65) | 422 | calcium; cell junction; cytoplasmic vesicle; dimer; duplication; glycoprotein; lipoprotein; membrane; membrane trafficking; metal-binding; palmitate; phospholipid binding; phosphorylation; repeat; synapse; synaptic vesicle; transmembrane; transmembrane pr | C2 domain |
| O00445 | SYT5_HUMAN | Synaptotagmin-5 (Synaptotagmin V) (SytV) | 386 | calcium; cell junction; cytoplasmic vesicle; endosome; membrane; metal-binding; polymorphism; repeat; synapse; transmembrane | C2 domain |
| Q86SS6 | SYT9_HUMAN | Synaptotagmin-9 (Synaptotagmin IX) (SytIX) | 491 | calcium; cell junction; cytoplasmic vesicle; membrane; metal-binding; repeat; synapse; transmembrane | C2 domain |
| Q96C24 | SYTL4_HUMAN | Synaptotagmin-like protein 4 (Exophilin-2) (Granuphilin) | 671 | 3d-structure; alternative splicing; membrane; metal-binding; phosphorylation; polymorphism; repeat; zinc; zinc-finger | C2 domain; Rabphilin-3A effector domain |
| Q8TDW5 | SYTL5_HUMAN | Synaptotagmin-like protein 5 | 730 | membrane; metal-binding; polymorphism; repeat; zinc; zinc-finger | C2 domain; Rabphilin-3A effector domain |
| Q8TC26 | TM163_HUMAN | Transmembrane protein 163 | 289 | alternative splicing; membrane; phosphorylation; transmembrane |  |
| Q9Y5R8 | TPPC1_HUMAN | Trafficking protein particle complex subunit 1 (BET5 homolog) (Multiple myeloma protein 2) (MUM-2) | 145 | disease mutation; endoplasmic reticulum; er-golgi transport; golgi apparatus; transport | Sybindin-like family |
| Q9Y296 | TPPC4_HUMAN | Trafficking protein particle complex subunit 4 (Synbindin) (TRS23 homolog) (Hematopoietic stem/progenitor cell protein 172) | 219 | 3d-structure; endoplasmic reticulum; er-golgi transport; golgi apparatus; transport | Sybindin-like family |
| O95859 | TSN12_HUMAN | Tetraspanin-12 (Tspan-12) (Transmembrane 4 superfamily member 12) (Tetraspan NET-2) | 305 | membrane; transmembrane | Tetraspanin family |
| P40222 | TXLNA_HUMAN | Alpha-taxilin | 546 | B-cell; B-cell lymphoma; T-cell; **coiled** coil; cytokine; exocytosis; extracellular protein; glycoprotein; growth factor; phosphorylation |  |
| O14795 | UN13B_HUMAN | Unc-13 homolog B (Munc13-2) (munc13) | 1591 | cell junction; **coiled** coil; cytoplasm; exocytosis; membrane; metal-binding; phorbol-ester binding; repeat; synapse; zinc; zinc-finger | Phorbol esters/diacylglycerol binding domain; C2 domain; Domain of Unknown Function |
| Q70J99 | UN13D_HUMAN | Unc-13 homolog D (Munc13-4) | 1090 | alternative splicing; cytoplasm; exocytosis; familial hemophagocytic lymphohistiocytosis; membrane; polymorphism; repeat | C2 domain; Domain of Unknown Function |
| P63027 | VAMP2_HUMAN | Vesicle-associated membrane protein 2 (VAMP-2) (Synaptobrevin-2) | 116 | 3d-structure; acetylation; cell junction; **coiled** coil; cytoplasmic vesicle; membrane; membrane trafficking; phosphorylation; synapse; synaptosome; transmembrane; transmembrane protein | Synaptobrevin |
| Q9P0L0 | VAPA_HUMAN | Vesicle-associated membrane protein-associated protein A (VAMP- associated protein A) (VAMP-A) (VAP-A) (33 kDa Vamp-associated protein) (VAP-33) | 249 | **coiled** coil; membrane; transmembrane | MSP |
| Q99536 | VAT1_HUMAN | Synaptic vesicle membrane protein VAT-1 homolog (EC 1.-.-.-) | 393 | oxidoreductase | Zinc-binding dehydrogenase; Alcohol dehydrogenase GroES-like domain |
| P27449 | VATL_HUMAN | Vacuolar ATP synthase 16 kDa proteolipid subunit (EC 3.6.3.14) | 155 | ATP; atp synthesis; host-virus interaction; hydrogen ion transport; hydrolase; ion transport; membrane; transmembrane; transmembrane protein; transport; vacuole | ATP synthase subunit C |
| Q9H267 | VP33B_HUMAN | Vacuolar protein sorting-associated protein 33B (hVPS33B) | 617 | disease mutation; endosome; lysosome; membrane; polymorphism; protein transport; transport | Sec1 family |
| Q9P253 | VPS18_HUMAN | Vacuolar protein sorting-associated protein 18 homolog (hVPS18) | 973 | alternative splicing; **coiled** coil; endosome; lysosome; membrane; metal-binding; phosphorylation; protein transport; transport; zinc; zinc-finger | Pep3/Vps18/deep orange family |
| Q9UEU0 | VTI1B_HUMAN | Vesicle transport through interaction with t-SNAREs homolog 1B (Vesicle transport v-SNARE protein Vti1-like 1) (Vti1-rp1) | 232 | alternative splicing; **coiled** coil; membrane; protein transport; transmembrane; transport | Vesicle transport v-SNARE protein |

**Additional data file 2**

PS120, a list of presynaptic protein prototypes in human in view of their homologues in insects: fly, beetle, mosquito and honeybee. The protein list is compiled as described in Material and methods and are strongly supported by literature and experimental data. **(A)** The similarity (S) and identity (I) in sequence for the aligned sequence are indicated. Protein lengths are indicated in all cases that difference in length between human and insect homolog is greater than 10%. SIM indicates the similarity levels marked as A-C. Proteins with sequence similarity <64% are marked by C; proteins that share >65% and >75% similarity are marked by B and A, respectively. The missing genes (mis) in all of some of the insects are marked by an asterisk. Cell’ and ‘proc.’ denote the cellular localization and the dominating process of the protein in the synapse. The cellular processes are divided to ST, signal transduction, Cyto, cytoskeleton; M, integral membranous; EZ, enzymatic activity. Empty cells indicate that cases in which dominating assignment can not be provided. Assignments are based on manual expert annotations. **(B)** The PS120 according to human representatives and based on UniProt annotations including Pfam domains, protein length and functional assignment by SwissProt keyword. Proteins with coiled coil regions and proteins with C2 domains are underlined .
