# Supplementary material for: Evolution of insect proteomes: insights into synapse organization and synaptic vesicle life cycle
Source: Genome Biol. 2008 Feb 7;9(2):R27. doi: 10.1186/gb-2008-9-2-r27 (PMC2374702; doi:10.1186/gb-2008-9-2-r27)
Supplement: Additional data file 3 — Listed are the 44 genes that had no clear homologs in certain insects. After homology search in the nucleotide databases, the majority could be accounted for as missed annotated genes. [file gb-2008-9-2-r27-S3.doc]

**Additional data file 3**

|  | **GENE** | **Comments and Sequence similarity search** |
| --- | --- | --- |
|  | APBA2 | **Beetle:** XM_001122064.1. Similar to CG32677-PA (LOC726317), Partial mRNA=435  E = 2e-36; I = 55%; S = 70% |
|  | ARFGEF2 | **Honeybee:** XR_014979.1. Similar to CG7578-PA, isoform A(LOC409370), mRNA=4919  E = 0.0; I=60%, S = 73% |
|  | BAIAP3 | **Honeybee:** XM_392442.3. Similar to unc-13-4A CG32381-PA (LOC408914), mRNA =2233  E = 2e-58 I =34%, P = 54% |
|  | BIN1 | **Beetle:** XM_001121406.1. Similar to bridging integrator 3 (LOC725583), mRNA =729  E = 2e-11; I =25%, S= 44% |
|  | BLOC1S1 | **Beetle:** similar to CG31064-PB, isoform B. **Not a real homolog** |
|  | BSN | Not in Insects |
|  | CADPS | Not in Mosquitoes |
|  | CNO | **Honeybee:** XM_624426.2 similar to breast carcinoma amplified 4 isoform b (LOC552047), mRNA = 827  E = 2e-16, I = 33%, S = 60%  Weak in beetle |
|  | CPLX2 | Not in Insects – CPLX4 |
|  | EXOC8 | **Beetle**: XM_964819.1. Similar to CG8475-PA, isoform A (LOC658431), mRNA =60889  **Very long transcript – not real**. E = 5e-80, I = 33%, S = 51%  **Honeybee**: XR_015044.1. Similar to exocyst 84-kDa subunit (LOC411775, GB14153), mRNA =2218. E = 2e-45, I = 31%, S = 53% |
| 1. 1 | EXPH5 | Not in Insects |
|  | FLJ20366 ‎ | Not in Insects |
|  | GAP43 | Not in Insects |
| 1. 1 | GOPC | Not in Fly, Honeybee and Mosquito |
|  | LPHN1 | **Beetle**: XM_964282.1. Similar to CG8639-PA (LOC657847), mRNA =3999  E = 3e-123, I = 38%, S =57%  **Mosquito:** not conserved |
|  | MSS4 | Not found in Honeybee |
|  | MUTED | Very weak to Beetle, Not in Mosquito  **Beetle**: similar to CG7071-PA, isoform A |
| 1. 1 | MYRIP | Not in Insects |
| 1. 1 | PACSIN1 | **Beetle:** CM000279 Length = 13894384  E = 7.72364e-45; I = 45%. Missed annotation |
| 1. 1 | PCLO | Not found in Insects |
| 1. 2 | PLDN | Not found in Honeybee |
| 1. . | PPFIA3 | XR_015009.1. Similar to Liprin- CG11199-PA, isoform A (LOC409902), mRNA =3564  E = 0.0, I = 47%, S = 60%  **Honeybee:** Group14.20, Length = 115121. Missed annotation. |
| 1. 2 | RAB3GAP | Not found in Honeybee |
| 1. . | RAB3IL1 | Not found in Mosquito and Fly |
|  | RABGAP1 | **Honeybee:** XM_392340.3 similar to Gef26 CG9491-PA (LOC408810), mRNA =4047  E = 3e-42, I = 35%, S= 55%. Missed annotation |
|  | RAPGEF4 | **Honeybee:** XM_624777.2 similar to CDC25 (LOC552404), partial mRNA=1177  E = 5e-25, I = 28%, S = 50%. Missed annotation |
| 1. 2 | RILP | **Honeybee**: XM_393625.3. Similar to CG11448-PA (LOC410142), mRNA =1173  E = 0.011 I = 27%.  Weak in Insects |
| 1. 2 | RIMBP2 | Appear in all insects. Similar to CG31302-PA. Weak similarity  Group2.40. Length = 85269. E = 1.6e-31, I = 38%. Missed annotations |
|  | SCIN | **Beetle**: XM_963211.1. Similar to Flightless-I protein (LOC656702), mRNA =3720  E = 6e-67, I = 27%, S = 45%. Missed annotation  **Honeybee:** XM_393805.3. Similar to quail CG6433-PA (LOC410324), mRNA =2796  E = 6e-65, I = 28%, S = 45%. Missed annotation |
|  | SNAPAP | **Beetle:** CM000282 Length = 17478683  Expect = 4.45563e-20, I = 48%. Missed annotation |
|  | SNIP | **Beetle**: similar to CG32809-PD**. Very weak in Beetle**  But found in fly, honeybee and mosquitoes |
|  | SNPH | Not in insect s, not homolog. Weak to dynactin, liprin like |
|  | SNX9 | **Beetle**: CM000279 Length = 13894384  E = 3.24581e-60, I = 36%. Missed annotation |
|  | STXBP6 | No Insect homolog, Weak to Exocyst Sec3 |
|  | SYNGR1 | **Honeybee:** XR_015081.1. Hypothetical LOC552402 (LOC552402), mRNA =704  E = 1e-15, I = 47%, S = 56%. Missed annotation |
|  | SYNPR | **Beetle**: Remote to XM_967892.1. Similar to synaptoporin (LOC661749), mRNA=1212  E = 4e-26, I = 42%, S = 58%. **Not in other insects** |
|  | SYP | **Beetle**: XM_967892.1 similar to synaptoporin (LOC661749), mRNA =1212  E = 2e-27, I = 38%, S = 54%. **Not in other insects** |
|  | SYT9 | Exist in all insects but mostly **conserved in the C2 domain region** |
|  | SYTL4 | Exist in all insects but mostly **conserved in the C2 domain region** |
|  | SYTL5 | Exist in all insects but mostly **conserved in the C2 domain region** |
| 1. 2 | TMEM163 | Not in insects |
|  | TXLNA | Not in Honeybee |
|  | UNC13B | **Honeybee:** Group6.26, Length = 539090  E = 1.00287e-103, I = 71%. Missed annotation. Homologues in all Insects to UNC13D |
|  | VAT-1 | Very weak in Fly |

**Additional data file 3**

Genome annotations and homology detection. Genes that were missing (as detailed in Additional data file 2A) in at least one insect are listed. BLAST search against the original assembled sequences was performed using tBlastn program (protein sequence against the translated nucleotides database). For 70% of this list, the gene or the gene annotation was not reported as "supported mRNA is missing". No attempt to finalize the missed annotation genes was made but the supporting evidence is provided. For additional 9 of the gene products in PS120 list, no homologs could be reliably assigned in the official gene lists of multiple insects.
