# Supplementary material for: Evolution of insect proteomes: insights into synapse organization and synaptic vesicle life cycle
Source: Genome Biol. 2008 Feb 7;9(2):R27. doi: 10.1186/gb-2008-9-2-r27 (PMC2374702; doi:10.1186/gb-2008-9-2-r27)
Supplement: Additional data file 7 — Provided is a detailed table of the PS120 gene set with the level of sequence identity and similarity (expressed as percentage between human and insects) and the protein valence according to the STRING tool. [file gb-2008-9-2-r27-S7.doc]

**Additional data file 6**

| **Gene**  **Symbol** | **Identity**  **(%)** | **Similarity**  **(%)** | **No. of Partners** |
| --- | --- | --- | --- |
| BSN | 0 | 0 | 0 |
| FLJ20366 ‎ | 0 | 0 | 0 |
| GAP43 | 0 | 0 | 3 |
| MYRIP | 0 | 0 | 3 |
| RILP | 0 | 0 | 0 |
| SNPH | 0 | 0 | 2 |
| SYNPR | 0 | 0 | 0 |
| SYP | 0 | 0 | 10 |
| TMEM163 | 0 | 0 | 0 |
| EXPH5 | 21 | 40 | 0 |
| STXBP6 | 24 | 47 | 1 |
| GMRP | 25 | 38 | 10 |
| LAMP1 | 26 | 44 | 1 |
| PCLO | 27 | 40 | 0 |
| PLDN | 27 | 48 | 7 |
| CNTNAP1 | 29 | 47 | 8 |
| SALF | 29 | 47 | 2 |
| UNC13D | 29 | 46 | 3 |
| RAB3GAP | 30 | 49 | 2 |
| VTI1B | 30 | 55 | 8 |
| CNO | 31 | 51 | 3 |
| SV2A | 31 | 51 | 0 |
| VPS33B | 31 | 51 | 1 |
| EPS15 | 32 | 46 | 22 |
| MUTED | 32 | 51 | 0 |
| SNIP | 32 | 52 | 3 |
| AMPH | 33 | 53 | 6 |
| ITSN2 | 33 | 50 | 0 |
| NLGN2‎ | 33 | 50 | 3 |
| BAIAP3 | 33 | 49 | 1 |
| VPS18 | 34 | 54 | 3 |
| SNAP29 | 34 | 54 | 5 |
| ADD2 | 35 | 56 | 1 |
| RIMBP2 | 35 | 54 | 0 |
| SNX9 | 35 | 54 | 8 |
| BIN1 | 36 | 53 | 12 |
| NRXN1 | 36 | 55 | 5 |
| ERC1 | 37 | 52 | 7 |
| RIMS1 | 37 | 50 | 6 |
| SIPA1L1 | 37 | 52 | 1 |
| SNAP91 | 37 | 49 | 4 |
| LPHN1 | 38 | 55 | 2 |
| SYTL5 | 38 | 52 | 0 |
| TXLNA | 40 | 65 | 0 |
| NET2 | 41 | 59 | 1 |
| RAB6IP1 | 41 | 57 | 1 |
| RPH3A | 41 | 63 | 5 |
| MSS4 | 42 | 53 | 1 |
| PACSIN1 | 42 | 61 | 2 |
| SCIN | 42 | 59 | 1 |
| SLC17A7 | 42 | 62 | 0 |
| CPLX2 | 43 | 59 | 0 |
| DOC2B | 43 | 60 | 1 |
| EPS1 | 43 | 56 | 11 |
| RAB3IL1 | 43 | 62 | 0 |
| SYN1 | 43 | 61 | 8 |
| SYTL4 | 43 | 61 | 7 |
| VAPA | 43 | 65 | 4 |
| BLOC1S2 | 44 | 61 | 6 |
| RABAC1 | 44 | 66 | 7 |
| HGS | 45 | 56 | 7 |
| PICALM | 45 | 58 | 7 |
| VAT1 | 46 | 63 | 0 |
| SCAMP1 | 47 | 62 | 3 |
| GOPC | 48 | 66 | 5 |
| GOSR2 | 48 | 69 | 13 |
| RABGAP1 | 48 | 69 | 0 |
| SNAPAP | 48 | 72 | 10 |
| STXBP5 | 48 | 65 | 0 |
| APBA1 | 49 | 63 | 8 |
| PIK4CA | 49 | 66 | 10 |
| EXOC6 | 51 | 70 | 6 |
| RAPGEF4 | 51 | 68 | 4 |
| SYNGR1 | 51 | 66 | 0 |
| BET1 | 52 | 62 | 5 |
| SH3GL1 | 53 | 69 | 4 |
| SYNJ1 | 53 | 68 | 9 |
| SYT9 | 53 | 69 | 1 |
| CADPS | 54 | 69 | 0 |
| TRAPPC1 | 54 | 79 | 1 |
| PIP5K1C | 55 | 67 | 1 |
| CACNA1A | 57 | 73 | 12 |
| SYT5 | 58 | 77 | 0 |
| DLG1 | 59 | 74 | 1 |
| DNAJC5 | 59 | 74 | 1 |
| KIF1A | 59 | 73 | 7 |
| SYBL1 | 59 | 78 | 7 |
| APBA2 | 60 | 74 | 2 |
| SNAP25 | 61 | 77 | 28 |
| TRAPPC4 | 61 | 80 | 1 |
| ARFGEF2 | 61 | 74 | 1 |
| SEPT5 | 62 | 79 | 5 |
| SEC22B | 63 | 78 | 3 |
| ARFIP2 | 63 | 80 | 6 |
| PPFIA3‎ | 63 | 80 | 0 |
| SYT1 | 64 | 78 | 47 |
| NSF | 65 | 80 | 19 |
| CASK | 66 | 79 | 11 |
| GDI2 | 66 | 82 | 7 |
| SNAPA | 66 | 84 | 8 |
| STXBP1 | 66 | 79 | 7 |
| EHD1 | 67 | 82 | 3 |
| PSCD2 | 67 | 83 | 3 |
| UNC13B | 68 | 81 | 8 |
| RAB27A | 69 | 84 | 13 |
| AP3D1 | 70 | 86 | 6 |
| PSCD1 | 70 | 85 | 2 |
| RALA | 70 | 80 | 14 |
| AP2A1 | 71 | 82 | 20 |
| VAMP2 | 72 | 80 | 20 |
| DNM1 | 73 | 89 | 23 |
| STX1A | 73 | 86 | 20 |
| YWHAQ | 76 | 86 | 4 |
| LIN7A | 79 | 90 | 6 |
| RAB3A | 80 | 90 | 10 |
| CLTC | 81 | 90 | 9 |
| ATP6V0C | 82 | 89 | 1 |
| ARF1 | 95 | 99 | 37 |
| CALM2 | 97 | 99 | 50 |
| ARF6 | 97 | 100 | 13 |

**Additional data file 6**

A complete list of the PS120 genes according to the conservation index as reflected by the identity and similarity (%) between human and the most conserved insects among the 4 representatives: honeybee, mosquito, fly and beetle. The number of interacting partners for each protein is determined by the String tool with the following setting - score >0.9 and maximal number of interacting proteins is limited to 50 (see Materials and methods).
