# Supplementary material for: Evolution of insect proteomes: insights into synapse organization and synaptic vesicle life cycle
Source: Genome Biol. 2008 Feb 7;9(2):R27. doi: 10.1186/gb-2008-9-2-r27 (PMC2374702; doi:10.1186/gb-2008-9-2-r27)
Supplement: Additional data file 8 — Presented is a detailed table of gene functions in endocytosis and exocytosis (24 genes each). Information on the number of interacting proteins, level of sequence identity between human and insect, and additional structural properties is included. [file gb-2008-9-2-r27-S8.doc]

**Additional data file 8**

| **ENDOCYTOSIS** | **Identity** | **Similarity** | **partners** | **Mem** | **Length aa** |
| --- | --- | --- | --- | --- | --- |
| AMPH | 33 | 53 | 6 |  | 695 |
| AP2A1 | 71 | 82 | 20 |  | 977 |
| AP3D1 | 70 | 86 | 6 |  | 1153 |
| ARF6 | 97 | 100 | 13 |  | 175 |
| ARFGEF2 | 61 | 74 | 1 |  | 1785 |
| ARFIP2 | 63 | 80 | 6 |  | 341 |
| BIN | 36 | 53 | 12 |  | 593 |
| CLTC | 81 | 90 | 9 |  | 1675 |
| DNM1 | 73 | 89 | 23 |  | 736 |
| EHD1 | 67 | 82 | 3 |  | 534 |
| EPN1 | 43 | 56 | 11 |  | 551 |
| EPS15 | 32 | 46 | 22 |  | 896 |
| HGS | 45 | 56 | 7 |  | 777 |
| ITSN2 | 33 | 50 | 0 |  | 1696 |
| PACSIN1 | 42 | 61 | 2 |  | 444 |
| PICALM | 45 | 58 | 7 |  | 652 |
| PIK4CA | 49 | 66 | 10 |  | 854 |
| PIP5K1C | 55 | 67 | 1 |  | 668 |
| SALF | 29 | 47 | 2 |  | 735 |
| SH3GL1 | 53 | 69 | 4 |  | 353 |
| SNAP91 | 37 | 49 | 4 |  | 907 |
| SNX9 | 35 | 54 | 8 |  | 595 |
| SYNJ1 | 53 | 68 | 9 |  | 1575 |
| VPS18 | 34 | 54 | 3 |  | 973 |
| **Average** |  |  | **7.9** |  | **847** |
| **EXOCYTOSIS** | **Identity** | **Similarity** | **partners** | **Mem** | **Length aa** |
| ATP6V0C | 82 | 89 | 1 | M | 155 |
| BET1 | 52 | 62 | 5 | M | 118 |
| CACNA1A | 57 | 73 | 12 | M | 980 |
| CPLX2 | 43 | 59 | 0 |  | 139 |
| GOSR2 | 48 | 69 | 13 |  | 212 |
| MSS4 | 42 | 53 | 1 |  | 123 |
| NSF | 65 | 80 | 19 |  | 744 |
| RAB27A | 69 | 84 | 13 | L | 221 |
| RAB3A | 80 | 90 | 10 | L | 220 |
| SEC22B | 63 | 78 | 3 | M | 215 |
| SNAP25 | 61 | 77 | 28 | L | 206 |
| SNAP29 | 34 | 54 | 5 | L | 258 |
| SNAPA | 66 | 84 | 8 |  | 295 |
| SNAPAP | 48 | 72 | 10 |  | 136 |
| SNIP | 32 | 52 | 3 |  | 1055 |
| STX1A | 73 | 86 | 20 | M | 288 |
| STXBP1 | 66 | 79 | 7 |  | 594 |
| STXBP6 | 24 | 47 | 1 |  | 210 |
| SV2A | 31 | 51 | 0 | M | 742 |
| SYT1 | 64 | 78 | 47 | M | 422 |
| TRAPPC1 | 54 | 79 | 1 |  | 145 |
| VAMP2 | 72 | 80 | 20 | M | 116 |
| VAPA | 43 | 65 | 4 | M | 249 |
| VTI1B | 30 | 55 | 8 | M | 232 |
| **Average** |  |  | **10.0** |  | **336** |

**Additional data file 8**

Two non-overlapping of exocytotic and endocytotic proteins (24 proteins each) are listed. Each protein is associated with the degree of conservation (according to the % similarity and identity between human and insect sequences), the number of protein partners according to String tool with score >0.9 and the protein length (in amino acids, aa). Mem, membrane localization is indicated where in ‘M’ are the proteins with at least one TMD and Marked as ‘L’ indicate those that are integral to the membrane following a lipid modification step.
